# Bioinformatic, Enzymatic, and Structural Characterization of *Trichuris suis* Hexosaminidase HEX-2

**DOI:** 10.1021/acs.biochem.4c00187

**Published:** 2024-07-26

**Authors:** Zuzanna Dutkiewicz, Annabelle Varrot, Karen J. Breese, Keith A. Stubbs, Lena Nuschy, Isabella Adduci, Katharina Paschinger, Iain B. H. Wilson

**Affiliations:** † Institut für Biochemie, Department für Chemie, Universität für Bodenkultur, Muthgasse 18, Wien 1190, Austria; ‡ Univ. Grenoble Alpes, CNRS, CERMAV, Grenoble 38000, France; § School of Molecular Sciences, University of Western Australia, Crawley, WA 6009, Australia; ∥ ARC Training Centre for Next-Gen Technologies in Biomedical Analysis, School of Molecular Sciences, University of Western Australia, Crawley, WA 6009, Australia; ⊥ Institut für Parasitologie, Department für Pathobiologie, Veterinärmedizinische Universität Wien, Veterinärplatz 1, Wien A-1210, Austria

## Abstract

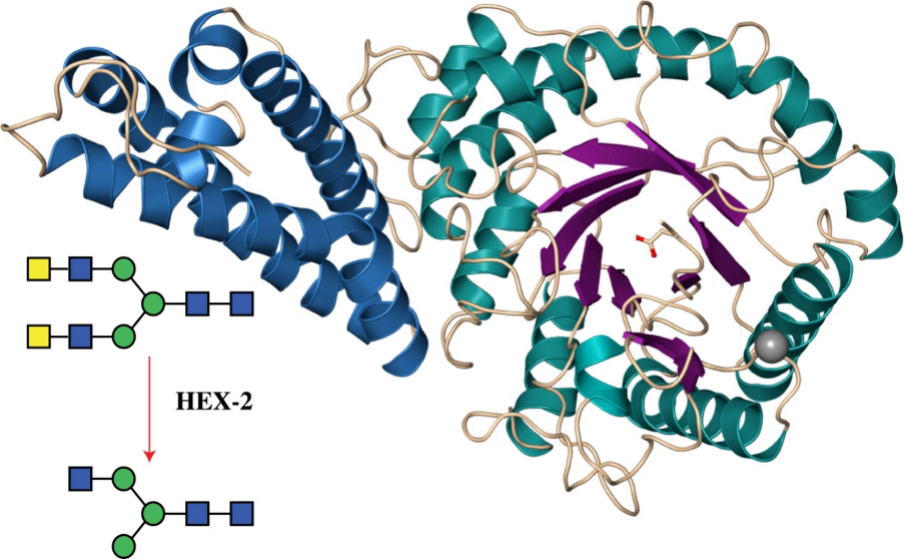

Hexosaminidases are key enzymes in glycoconjugate metabolism and occur in all kingdoms of life. Here, we have investigated the phylogeny of the GH20 glycosyl hydrolase family in nematodes and identified a β-hexosaminidase subclade present only in the Dorylaimia. We have expressed one of these, HEX-2 from *Trichuris suis*, a porcine parasite, and shown that it prefers an aryl β-*N*-acetylgalactosaminide *in vitro*. HEX-2 has an almost neutral pH optimum and is best inhibited by GalNAc-isofagomine. Toward N-glycan substrates, it displays a preference for the removal of GalNAc residues from LacdiNAc motifs as well as the GlcNAc attached to the α1,3-linked core mannose. Therefore, it has a broader specificity than insect fused lobe (FDL) hexosaminidases but one narrower than distant homologues from plants. Its X-ray crystal structure, the first of any subfamily 1 GH20 hexosaminidase to be determined, is closest to *Streptococcus pneumoniae* GH20C and the active site is predicted to be compatible with accommodating both GalNAc and GlcNAc. The new structure extends our knowledge about this large enzyme family, particularly as *T. suis* HEX-2 also possesses the key glutamate residue found in human hexosaminidases of either GH20 subfamily, including HEXD whose biological function remains elusive.

## Introduction

Hexosaminidases are enzymes ubiquitous across all domains of life and play multiple roles in glycoconjugate metabolism as they remove nonreducing terminal *N*-acetylgalactosamine and *N*-acetylglucosamine residues from glycans, glycolipids, glycoproteins, and glycosaminoglycans. In the case of β-hexosaminidases acting on nonreducing termini, most sequences are found in glycoside hydrolase families GH3 and GH20, which have distinct chemical mechanisms.^1^ Whereas β-hexosaminidases are primarily catabolic in mammals, in invertebrate species, they are often mediating purposeful processing steps, analogous to Golgi mannosidases, during the maturation of N-linked oligosaccharides.^2^ However, the biological significance of, and the structural basis for, hexosaminidase-mediated glycan-processing in nonvertebrates are poorly understood.

**Figure 1:**
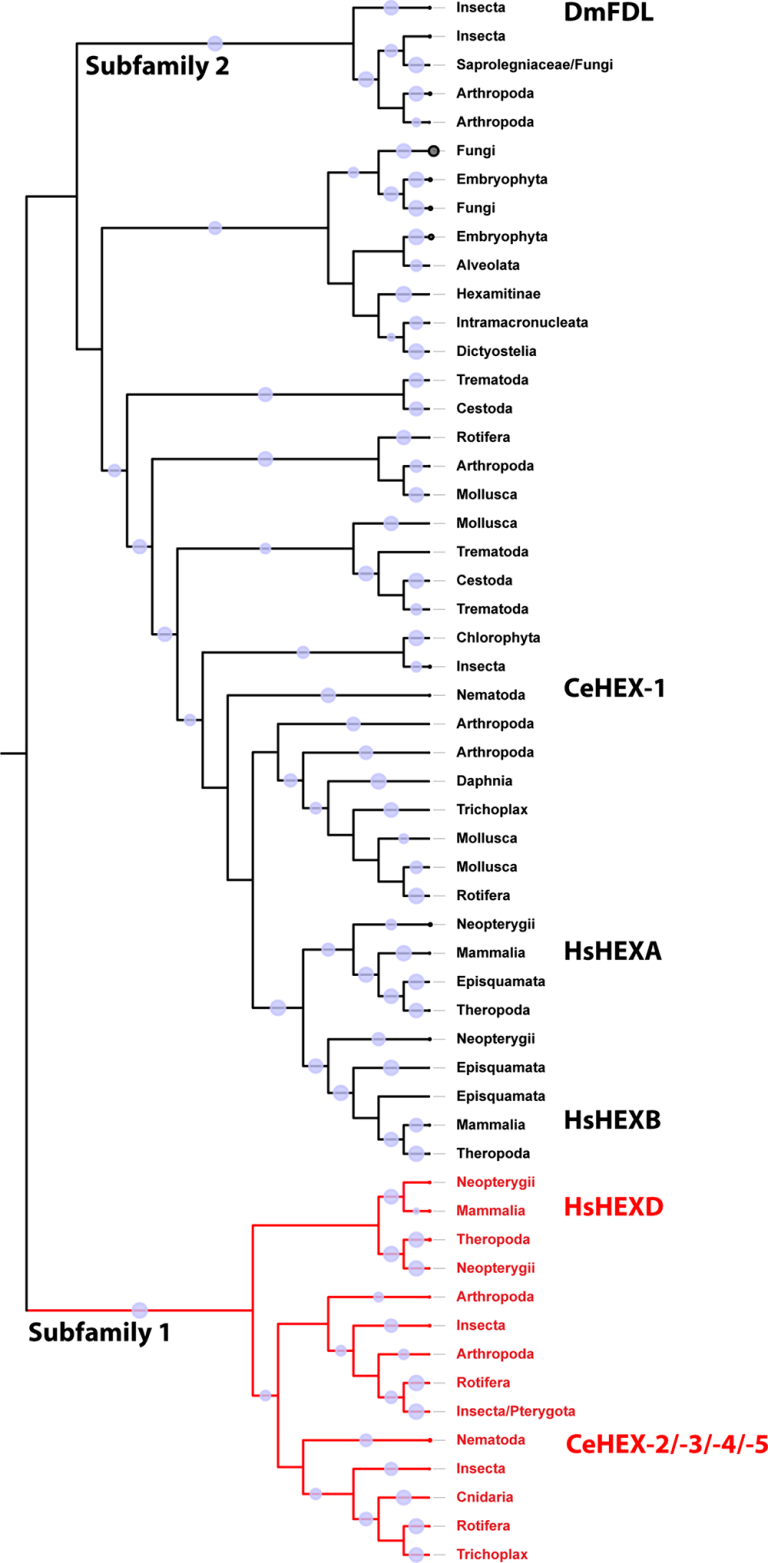
Phylogenic reconstruction of eukaryotic hexosaminidases. Subfamily 2 represents homologues of human HEXA and HEXB (HsHEXA and HsHEXB), *C. elegans* HEX-1 (CeHEX-1), and insect FDL (DmFDL); subfamily 1 (in red) contains homologues of human HEXD and *C. elegans* HEX-2/-3/-4/-5. Annotation was done based on known sequences from the literature. FastTree was used to generate an approximate maximum-likelihood phylogenetic tree, which was rooted at the midpoint. Blue circles represent bootstrap support between 70 and 100. Subfamilies 1 and 2 as categorized by Gutternigg et al.^2^ correspond to clades B and A defined by Intra et al.^26^

Four β-hexosaminidase genes are known from mammals: perhaps the most familiar are HEXA and HEXB encoding the α- and β-subunits of the hetero- and homodimeric lysosomal enzymes, which have been shown to be deficient in two storage diseases (Tay-Sachs and Sandhoff diseases, respectively),^3^ OGA encoding a nucleocytoplasmic O-GlcNAc-specific cleaving activity of family GH84 with roles in signaling^4^ and HEXDC encoding the nucleocytoplasmic hexosaminidase D, with an uncertain biological role. The latter enzyme^5,6^ is a member of GH20 subfamily 1 and is only distantly related to HEXA and HEXB, which are in the subfamily 2 (see Figure 1). Hexosaminidase D has a neutral pH optimum and preference for aryl *N*-acetyl-d-galactosaminides;^5−7^ this enzyme is apparently significantly responsible for elevated hexosaminidase activity in synovia in rheumatoid arthritis patients.^8^

When considering nonvertebrate species, among the best-described hexosaminidases are those from insects. For example, *Drosophila melanogaster* possesses a number of β-hexosaminidase genes: (i) the cytoplasmic OGA encoding the O-GlcNAc-specific enzyme similar to that in mammals,^9^ (ii) one GH20 subfamily member 1 (CG7985) with no characterized enzymatic function,^10^ but phylogenetically relatively “close” to hexosaminidase D and (iii) three members of GH20 subfamily 2 including two chitinolytic and/or broad spectrum enzymes and one N-glycan-specific hexosaminidase.^11^ The latter is encoded by the *fused lobes (fdl)* gene named due to the brain morphology defect in the corresponding fruitfly mutant; as enzymes, insect FDL hexosaminidases have a particular specificity for the nonreducing terminal β1,2-GlcNAc linked to the “lower arm” α-1,3-mannose of N-glycans,^11−16^ thereby removing the GlcNAc transferred by MGAT1 (*N-*acetylglucosaminyltransferase I). The molecular identification of FDL explained earlier work indicating that a special N-glycan-processing enzyme was present in insect cell microsomes.^17^

The other major invertebrate model organism, the nematode *Caenorhabditis elegans*, possesses six β-hexosaminidase genes, but with a different subfamily bias as compared to insects: four of the encoded hexosaminidases belong to GH20 subfamily 1 (HEX-2, -3, -4, and -5), one to subfamily 2 (HEX-1), and one is a proven OGA from GH84 family.^2,18^ Similar to insect FDL, HEX-2 and -3 have proven activity toward the β-1,2-linked GlcNAc on the lower arm of N-glycans,^2^ corresponding to a hexosaminidase activity found in *C. elegans* microsomes;^19^ additionally, HEX-2 can also cleave nonreducing terminal GalNAc, a property also demonstrated for HEX-4 and -5.^2,12^ HEX-1, on the other hand, is apparently chitinolytic and is close phylogenetically to human HEXA and HEXB.^2^ Our use of GFP-promoter constructs suggested different tissue expression patterns for the *C. elegans* hex genes,^2^ whereas HPLC/MS-based analyses of *hex-2*, *hex-2*;*hex-3*, and *hex-4* mutants showed an impact of their ablation on the N-glycome.^2,20,21^ Regarding other nematodes, there is little biochemical information regarding homologous enzymes, but a secreted N-glycan-digesting hexosaminidase from *Trichinella spiralis*, with no defined sequence, has been biochemically characterized^22^ and may be closest to *C. elegans* HEX-1 in terms of its properties.

Considering that *C. elegans* HEX-2 and HEX-3 are FDL-like in terms of their impact on the N-glycome, whereas HEX-4 is GalNAc-specific, we wished to explore the properties of further hexosaminidases from other nematode species. Preliminary database searching suggested that some nematodes have, like *C. elegans*, a number of GH20 subfamily 1 genes; for instance, *Oesophagostomum dentatum*, a clade V nematode like *C. elegans*, has at least HEX-2, -3, and -5 orthologues, whereas *Trichinella spiralis* and *Trichuris suis* (both clade I nematodes) appear to have only one subfamily 1 enzyme. On the other hand, *O. dentatum* lacks N-glycans with terminal GalNAc residues^23^ and wild-type *C. elegans* has very few;^21^ both species rather have chito-oligomer-based antennae for their most complex N-glycans. In contrast, *T. suis*(24) and *T. spiralis*(25) are rich in N-glycans containing terminal GalNAc motifs, which may indicate a difference in the hexosaminidase-dependent processing between clade I and V species. Therefore, a thorough phylogenetic analysis was performed and the GH20 subfamily 1 candidate enzyme from *T. suis* was expressed recombinantly, characterized, and successfully crystallized, yielding the first experimental structure of a eukaryotic subfamily 1 GH20 hexosaminidase.

## Results

### Phylogeny of GH20 Hexosaminidases

Initially, phylogenetic analyses were performed to recreate a comprehensive evolutionary pathway for GH20 hexosaminidases, based on using almost 4,000 sequences from eukaryotes. The results verify that there are two distinct groups of these hexosaminidases: subfamily 1 and subfamily 2 (Figure 1). Subfamily 2 encompasses the more familiar mammalian HEXA and HEXB as well as the insect FDL and nematode HEX-1 homologues. In subfamily 1, which includes mammalian HEXD, a clearly separated clade of nematode hexosaminidases was observed, which is represented in *C. elegans* by the previously characterized HEX-2, HEX-3, HEX-4, and HEX-5 enzymes.^2,12,21^ Each *C. elegans* hexosaminidase is within its own distinct group of related sequences from other nematodes.

**Figure 2:**
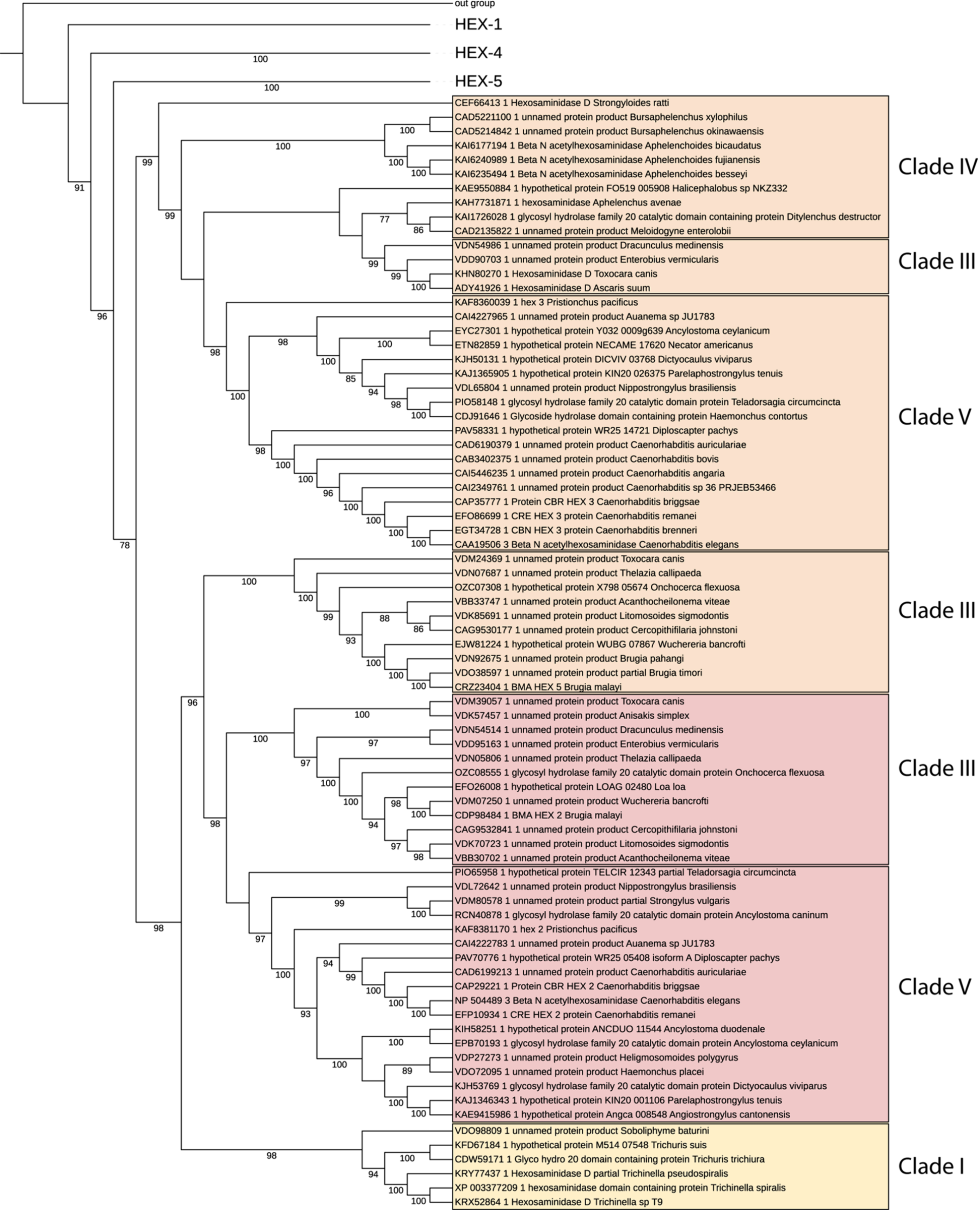
Nematode hexosaminidase phylogeny. Maximum likelihood (IQ-TREE) phylogeny of the nematode GH20 hexosaminidases. The HEX-2 and HEX-3 branches are highlighted and annotated with the groups of related species in terms of the Nematoda clades as defined by Blaxter;^27,28^Supplementary Figure S1A,B shows all the different nematode hexosaminidase branches. The *D. melanogaster* FDL sequence was used as an out group. Bootstrap values of >70 are shown. In the HEX-2 branch, the sequences highlighted in yellow represent a subclade of sequences in clade I species, which only have one subfamily 1 member each.

In *C. elegans*, the earliest separation occurs between HEX-4 and HEX-5 (Figure 2 and Supplementary Figure S1), indicating their specific ability to only remove GalNAc. Later, HEX-3 and HEX-2 evolved and are capable of removing both GalNAc and GlcNAc from glycan structures *in vitro*.^2^ The presence of numerous enzymes with similar functions indicates a significant amount of evolutionary pressure or possibly diverse applications for these enzymes. In general, the degree of relatedness within the GH20 clades correlates well with the proposed phylogeny of nematode species (e.g., filarial sequences are grouped together). Additionally, a subclade consisting of *Trichuris* spp. and *Trichinella* spp. representatives was identified in which only one hexosaminidase homologue per species, annotated as HEXD, was detected in the database. This finding is unusual compared to other nematodes that possess up to four subfamily 1 GH20 hexosaminidases but reflects that *Trichuris* spp. and *Trichinella* spp. are phylogenetically distinct from the majority of nematodes, falling within nematode clade I as defined by Blaxter.^27,28^ Based on phylogenetic analysis, it can be inferred that *Trichuris* and *Trichinella* enzymes are likely to have a similar activity to that of *C. elegans* HEX-2; thus, we designated the theoretical KFD87184 sequence as *T. suis* HEX-2. Overall, *T. suis* HEX-2 has around 40% identity over ca. 500 residues with *C. elegans* HEX-2 and HEX-3 (Supplementary Figure S2) and shares the His/Asn-Xaa-Gly-Yaa-Asp-Glu motif with many other GH20 hexosaminidases (Supplementary Figure S3), whereby this sequence is shifted toward the N-terminus of subfamily 1 sequences as compared to subfamily 2.

### Characterization of HEX-2

**Figure 3:**
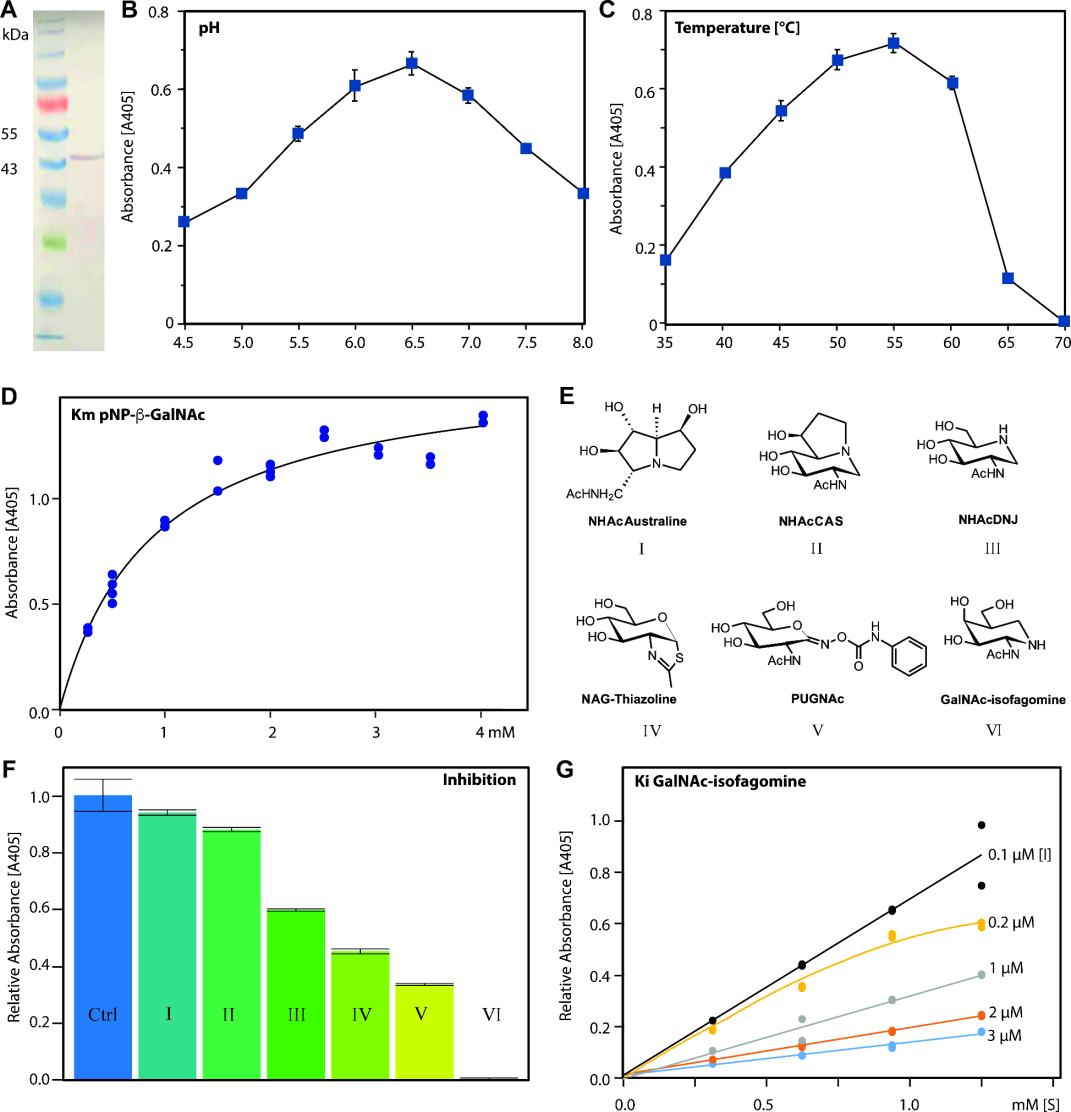
Activity of recombinant *T. suis* HEX-2 with a simple substrate. (A) Anti-His western blot of the purified recombinant C-terminally His6-tagged “short” form of *T. suis* HEX-2 expressed in *Pichia.* (B) pH dependency of activity toward pNP-β-GalNAc of recombinant *T. suis* HEX-2 assayed at 37 °C for 1 h using a range of McIlvaine buffers. (C) Temperature dependency of recombinant *T. suis* HEX-2. (D) Michaelis–Menten curve for *T. suis* HEX-2 with pNP-β-GalNAc as substrate. (E, F) Inhibition of *T. suis* HEX-2 protein using pNP-GalNAc as a substrate (5 mM) and six different competitive inhibitors (0.5 mM). (G) Graphical representation of the data obtained with GalNAc-isofagomine to calculate *K*_i_ as fitted by Prism (GraphPad). Each assay was performed in duplicate or triplicate and error bars indicate standard deviations; in panels (F) and (G), relative absorbance is in comparison to the activity of the uninhibited enzyme.

To determine whether *T. suis* HEX-2 had an activity similar to that of *C. elegans* HEX-2, we cloned the predicted open reading frame, excluding the sequence encoding the N-terminal cytoplasmic, transmembrane and stem domains (i.e., residues 85–620 or 137–620 of the predicted sequence, Supplementary Figure S2), into a *Pichia* vector for secreted expression. Only constructs with a C-terminal His-tag could be purified by immobilized metal affinity chromatography. Resulting ‘short’ or ‘long’ forms of the protein with apparent molecular weights of 50 or 75 kDa (Figure 3A and Supplementary Figure S4) were also verified by tryptic peptide mapping. In terms of enzymatic activity, we first examined the properties of *T. suis* HEX-2 using artificial aryl glycoside substrates. While pNP-β-GlcNAc was a poor substrate, there was excellent activity toward pNP-β-GalNAc (Supplementary Figure S4). It was observed that within the linear range of product formation with respect to time and in the presence of McIlvaine buffers, optimal activity was at pH 6–7 (Figure 3B), similar to the values for the *C. elegans* homologues HEX-2 and HEX-4.^26^ The optimal temperature was 50–60 °C (Figure 3C), similar to *C. elegans* HEX-2 and HEX-3.^12^ Incubation with a range of pNP-β-GalNAc substrate concentrations and 5 ng of *T. suis* HEX-2 allowed for the determination of an apparent *K*_m_ value of 0.9 mM (Figure 3D), which is also in the range for other characterized nematode hexosaminidases.

Six different known hexosaminidase inhibitors were tested (PUGNAc, NHAcDNJ, NHAcCAS, NHAc-Australine, NAG-Thiazoline, GalNAc-isofagomine,^29−34^Figure 3E) with *T. suis* HEX-2. After preincubation of the competitive inhibitors with the enzyme, pNP-β-GalNAc was again used as a substrate and the activity was determined. The highest degree of inhibition as compared to the control was observed with GalNAc-isofagomine and the least with NHAc-Australine (Figure 3F). The *K*_i_ for GalNAc-isofagomine was determined to be 0.6 μM (Figure 3G), a value lower than that determined for *Streptomyces plicatus* β-N-acetylhexosaminidase.^34^

### Specificity of *T. suis* HEX-2

**Figure 4:**
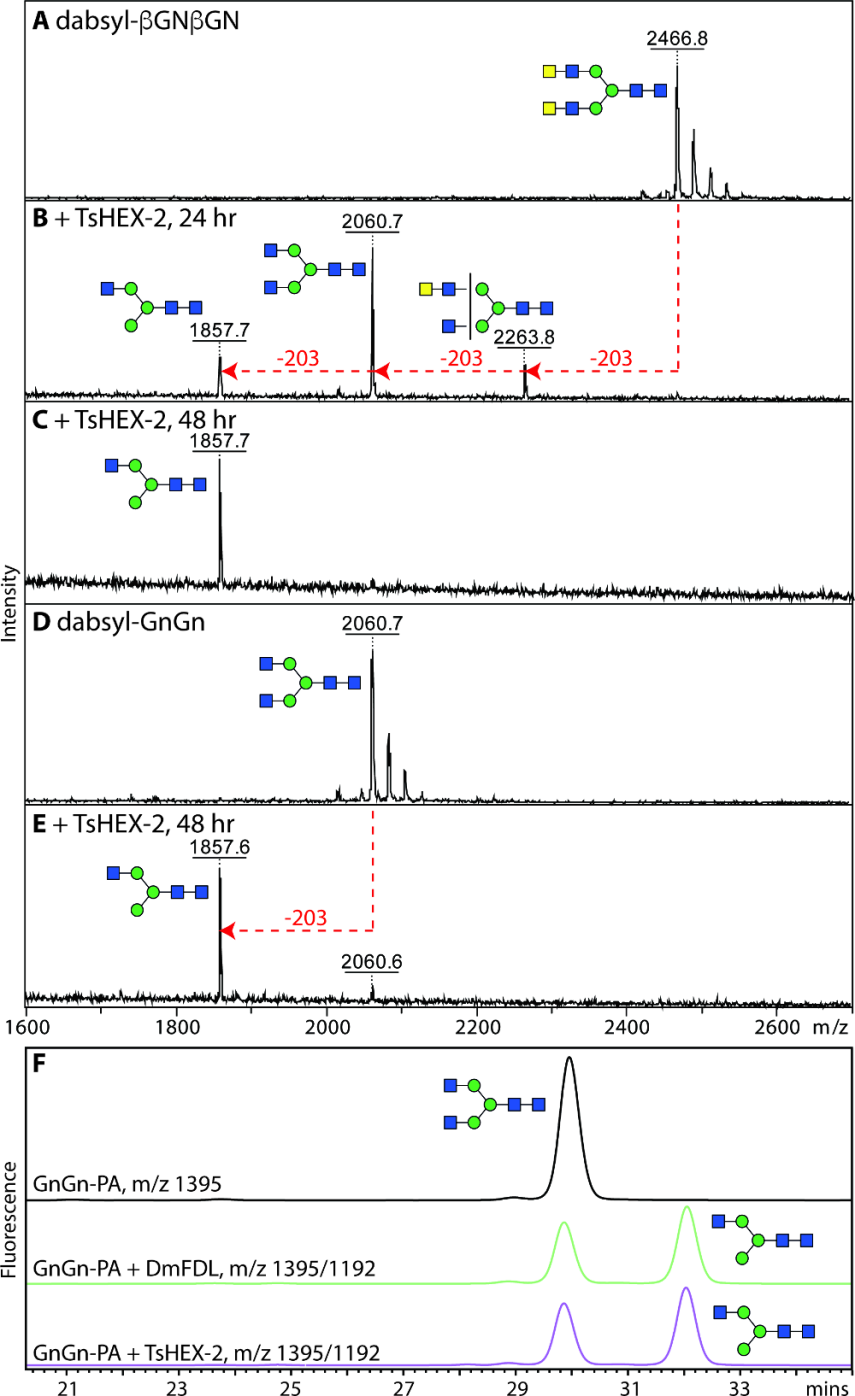
Activity of recombinant *T. suis* HEX-2 with biantennary glycan substrates. (A–E) MALDI-TOF MS analysis of incubations of dabsyl glycopeptides carrying either GnGn (*m*/*z* 2060) and βGNβGN (*m*/*z* 2467, with two LacdiNAc units) before (A/C) or after treatment with *T. suis* HEX-2 for 24 h (B), 48h (C/E). (F) RP-HPLC chromatogram of GnGn-PA (*m*/*z* 1395) before (black) or after treatment with either insect FDL (green) or *T. suis* HEX-2 (blue); a shift to later elution time is indicative of removal of the “lower” nonreducing terminal GlcNAc residue.^36^ Red lines with arrows indicate losses of HexNAc residues from the substrates. Glycans are depicted according to the Symbol Nomenclature for Glycans (SNFG).

**Figure 5:**
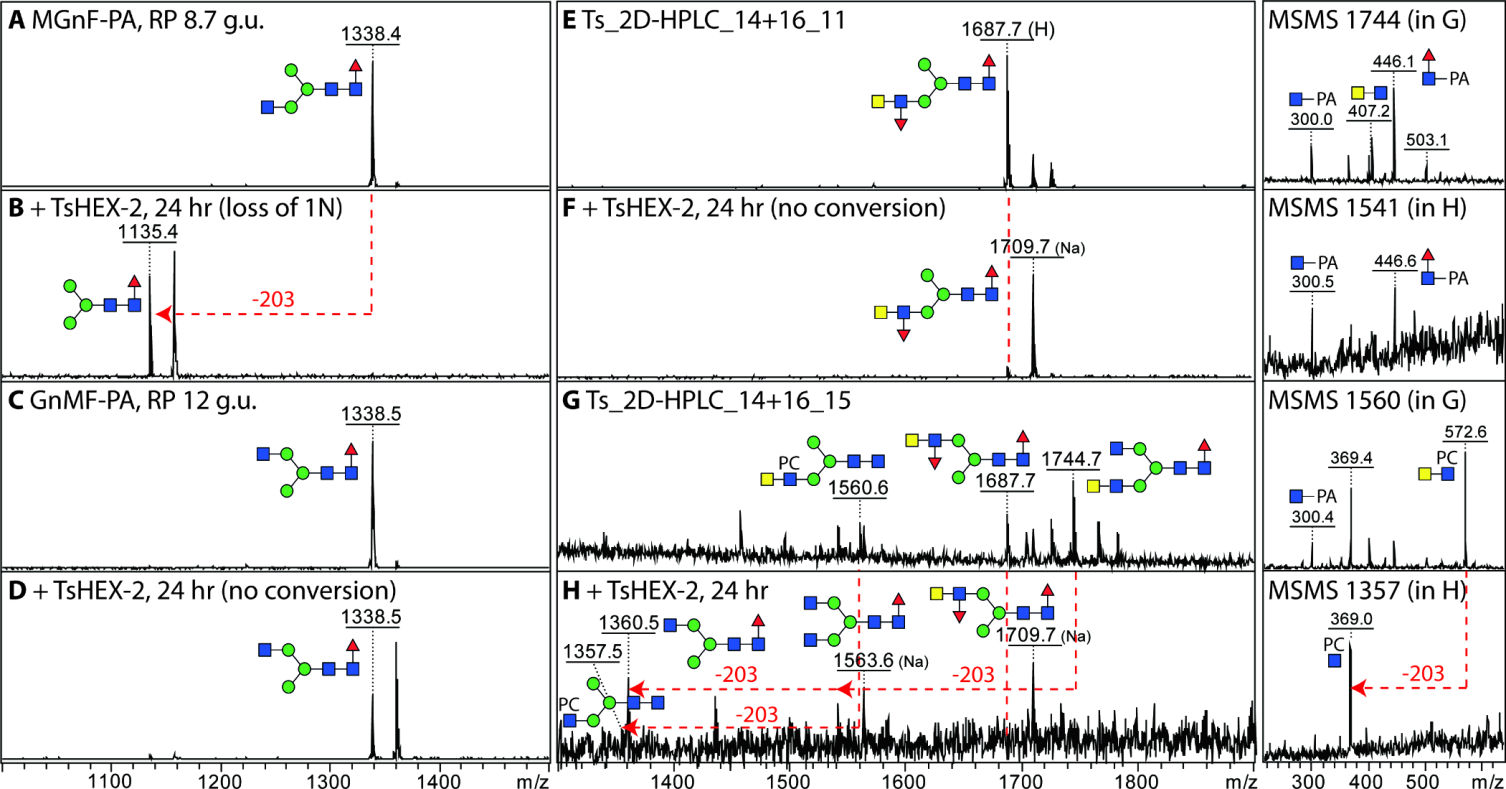
Activity of recombinant *T. suis* HEX-2 with complex nematode glycan substrates. (A–D) MALDI-TOF MS of *Dirofilaria immitis* glycans before (A/C) or after incubation with *T. suis* HEX-2 (B/D); while one Hex_3_HexNAc_3_Fuc isomer (MGnF, *m*/*z* 1338, eluting at 8.7 g.u. on RP-HPLC^35^) was sensitive (B), the second isomer (GnMF, eluting at 12 g.u.) was resistant. (E–H) MALDI-TOF MS of 2D-HPLC purified *T. suis* glycans before (E/G) and after incubation (F/H) with *T. suis* HEX-2; while two glycans with fucosylated LacdiNAc motifs (*m*/*z* 1687 as [M + H]^+^ or 1709 as [M + Na]^+^) were not digested, structures with either nonsubstituted or phosphorylcholine-substituted LacdiNAc (*m*/*z* 1744 and 1560 as [M + H]^+^) were sensitive to *T. suis* HEX-2 (respective products of *m*/*z* 1563 and 1360 as [M + Na]^+^ and 1357 as [M + H]^+^), resulting in alterations in the MS/MS spectra (loss of B-ion HexNAc_2_PC_0–1_ fragments of *m*/*z* 407 and 572). Note that the addition of HEX-2 results in a shift to sodiated ions for neutral glycans. Glycans are depicted according to the Symbol Nomenclature for Glycans (SNFG).

*T. suis* HEX*-*2 was tested with typical N-glycan substrates and shown to remove only one GlcNAc from a GnGn-dabsyl-N-glycopeptide (*m*/*z* 2060), but both GalNAc residues as well as just one terminal GlcNAc from a βGNβGN-dabsyl-N-glycopeptide (*m*/*z* 2467, with two LacdiNAc units) (Figure 4A–E). In order to test the arm specificity, RP-HPLC analysis of a pyridylamino-N-glycan (GnGn) before and after incubation with *T. suis* HEX-2 was performed; the shift to later retention time was indicative of removing solely the “lower” arm GlcNAc (Figure 4F) and dependent on the pH of the reaction mixture (Supplementary Figure S4D). Another test of the specificity was to take RP-HPLC-purified core fucosylated N-glycans from *Dirofilaria immitis* with either a lower or an upper arm GlcNAc;^35^ in the case of the former, the nonreducing terminal GlcNAc was removed (Figure 5A,B). In contrast, *T. suis* HEX-2 did not remove the upper arm of GlcNAc (Figure 5C,D). Regarding more complicated structures with LacdiNAc-based antennae, the activity of *T. suis* HEX-2 toward two selected N-glycan fractions derived from *T. suis* itself were selected. While the glycan with a fucosylated LacdiNAc was resistant to HEX-2 (Figure 5E,F), the structure with a phosphorylcholine-substituted LacdiNAc did lose the terminal GalNAc, as also reflected by the MS/MS data (Figure 5G,H)

### X-ray Crystallography

**Figure 6:**
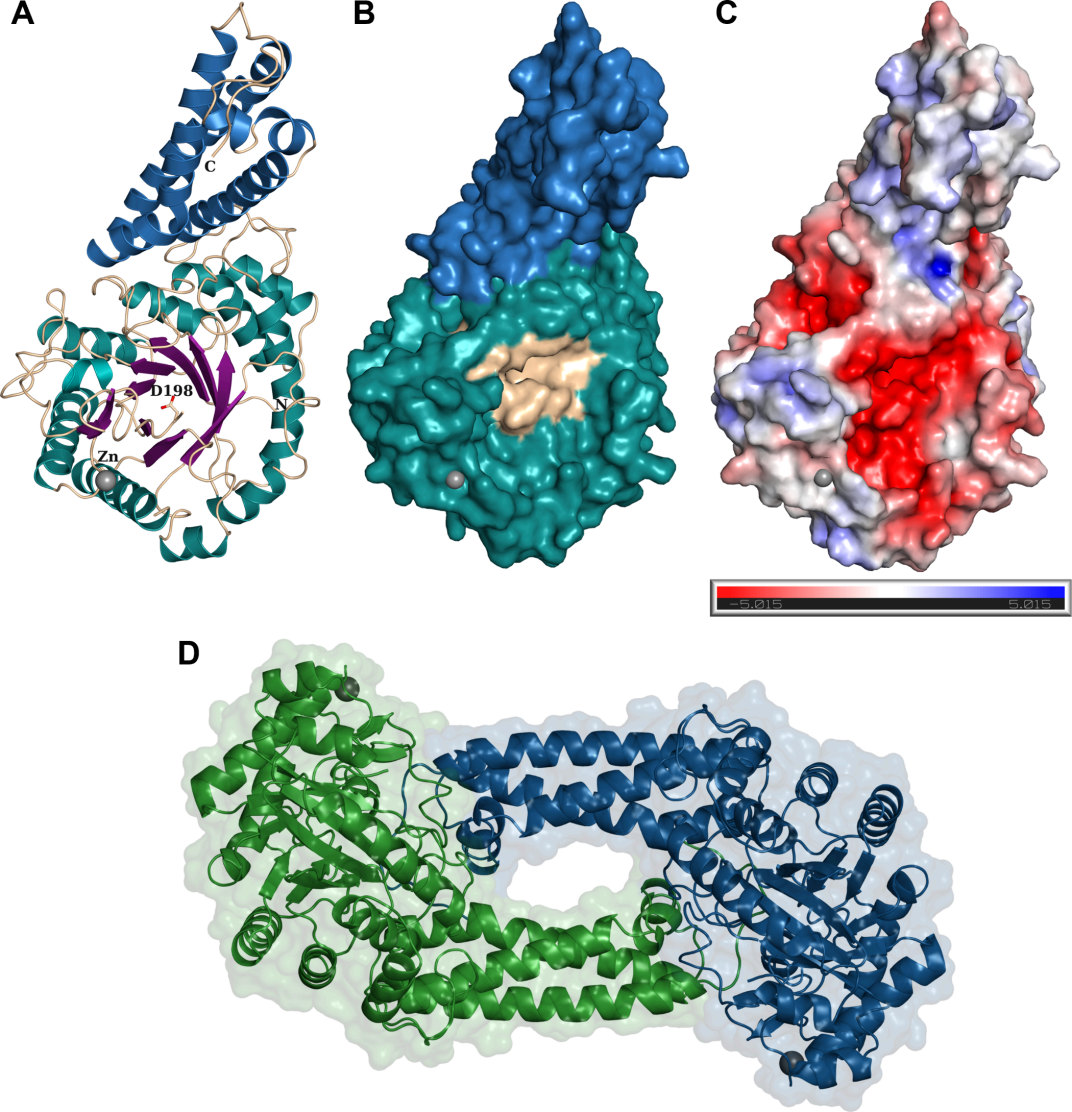
3D-structural analysis of recombinant *T. suis* HEX-2. (A) Cartoon representation of the X-ray crystal structure of *T. suis* HEX-2 at 2.55 Å. The TIM barrel and helix bundle are colored differently; the key catalytic Asp residue is represented as ball and sticks and Zn^2+^ ion as a gray ball. (B) Surface representation of HEX-2 with the helix bundle colored in blue, the TIM barrel in green and the active site area in wheat. (C) ±5 kT/e electrostatic potential of HEX-2 in PyMOL plotted on the solvent-accessible surface and calculated with APBS plugin.^40^ (D) Surface and cartoon representations of the HEX-2 potential dimer.

In order to gain insight into the specificity of *T. suis* HEX-2, we solved its X-ray crystal structure at a resolution of 2.55 Å in a *C*2 space group (Table 1). Overall, HEX-2 displays a modular structure with a N-terminal catalytic domain taking the shape of an (β/α)_8_ or TIM barrel followed by a C-terminal three helix bundle, whose function remains unknown. There is one protomer in the asymmetric unit (Figure 6A). Analysis of the interfaces and assemblies with PDBePISA^37^ revealed one interface leading to the formation of a crystallographic dimer around the 2-fold axis. The interface represents only 13% of the solvent accessible area (2610 Å) and includes 15.8% of the total residues located in surface loops of the C-terminal part of the three helices bundle interacting with the surface loops connecting strands and helices 2, 3, 7, and 8 of the TIM (Figure 6B). The complex formation significance score (CSS) of 0.3 indicates an auxiliary role of the interface in the dimer formation implying an unstable or weak dimer or a crystallographic artifact; other GH20 enzymes are indeed known to exist as dimers in solution, including murine HexD and OfHex1^6,38^ and HEX-2 migrated on native gel electrophoresis in multimeric forms (Supplementary Figure S4B). Although the enzyme is predicted to be N-glycosylated, no electron density for even a core GlcNAc could be detected. Disordered regions at the N- and C-terminus correspond to the probable stem and the hexahistidine tag, respectively. The structure also revealed the presence of a Zn^2+^ ion (Figure 6A) not far from the binding pocket. There is also a disordered surface loop between the Zn^2+^ site and the binding site which resulted in the lack of electron density between residues Glu199 and Arg220 so they could not be modeled (Supplementary Figure S5). Analysis of the closest related structure, the GH20C β-hexosaminidase from *Streptococcus pneumoniae*,^39^ indicates that this region should contain an α-helix. Additionally, the positioning of the Zn^2+^ ion implies a noncatalytic/structural role, potentially in the stabilization of this disordered loop upon substrate binding. Experiments indeed showed that up to 10 mM Zn(II) or EDTA had only minor effects on the enzyme activity (Supplementary Figure 4E,F). The N-terminal region of the crystallized protein with no observed electron density (residues 85–137 of the full theoretical KFD87184 sequence, corresponding to residues 1–53 of the construct) does not align well with *C. elegans* HEX-2 (Supplementary Figure S2) and was susceptible to proteolysis; as the “short” form of the enzyme lacking this region was active, it is assumed that the stem domain extends as far as Phe138 of the full theoretical sequence.

**Table 1:** Data Collection and Refinement Statistics^a^

**data collection**	
beamline	SOLEIL Proxima-1
wavelength	0.97856
space group	C2
unit cell dimensions	96.12 59.04 105.98 90.00 114.49 90.00
resolution (Å)	43.74–2.55 (2.66–2.55)
Nb reflections	116,794 (14,395)
Nb unique reflections	17,835 (2,157)
*R*_merge_	0.067 (0.782)
*R*_meas_	0.080 (0.931)
*R*_pim_	0.043 (0.500)
mean *I/*σ*I*	14.1 (2.1)
completeness (%)	99.9 (99.9)
redundancy	6.5 (6.7)
CC_1/2_	0.999 (0.843)

**refinement**	
resolution (Å)	43.74–2.55
no. reflections	17,268
no. free reflections	836
*R*_work_/*R*_free_	0.189/0.259
rmsd bond lengths (Å)	0.0120
rmsd bond angles (deg)	1.99
rmsd chiral (Å^3^)	0.0864
clashscore	11
No. atoms/Bfac (Å^2^)	
protein	3,688/44.5
water	45/32.3
Zn	1/81.3
ramachandran	
allowed/favored (%)	99.3/93.4
outliers	3
PDB Code	8QK1

a Values in parentheses are for the high-resolution shell.

**Figure 7:**
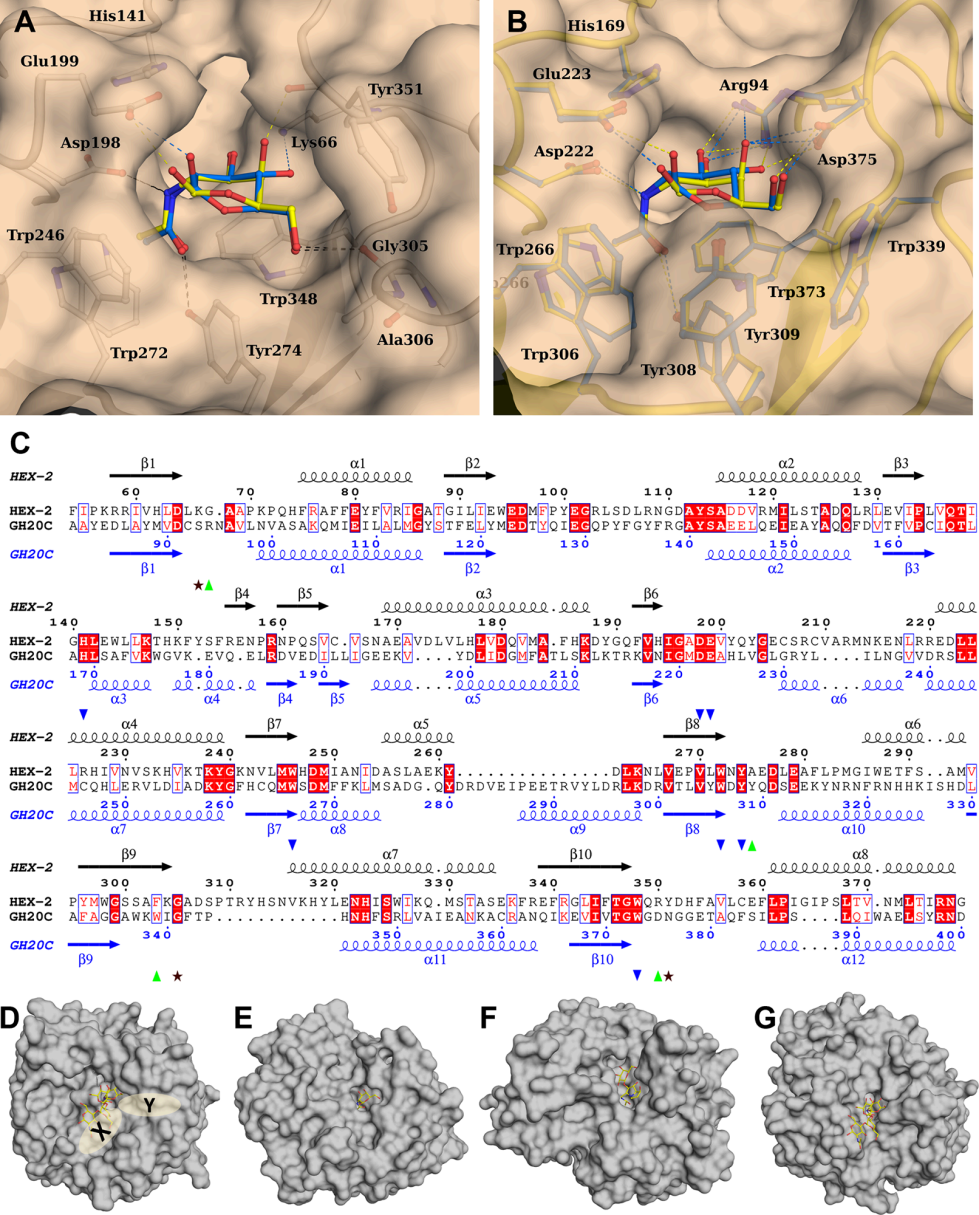
Modeling and alignments of the binding site pocket of the *T. suis* HEX-2 catalytic domain. (A) Interactions of HEX-2 with manually docked GalNAc (yellow) and GlcNAc (cyan). Position of Glu199 has been modified to the one expected upon binding. (B) Interactions of *S. pneumoniae* GH20C β-hexosaminidase with GalNAc (yellow carbons; PDB-ID: 5AC4) and GlcNAc (cyan carbons; PDB-ID: 5AC5). Amino acids are represented by balls and sticks; H-bonds are displayed in dash lines of corresponding color. (C) Alignment of HEX-2 with GH20-C highlighting the amino acids involved in the active site with the one conserved (blue triangles), those only found in HEX-2 (green triangles) and those only in GH20-C (brown stars). Alignment made by Clustal Omega^44^ and figure drawn with ESPript 3.0.^45^ Surface representation of the TIM barrel of *T. suis* HEX-2 (D), *S. pneumoniae* GH20C in complex with GlcNAc (E, PDB-ID: 5AC4), *Bifidobacterium bifidum* Lacto-*N*-biosidase LNBase in complex with LNB-NHAcAUS (F, PDB-ID: 5BXT) and *Enterococcus faecalis* endoglycosidase E GH20 domain (G, PDB-ID: 7PUL). Figures are drawn in the same orientation after overlay on the HEX-2 catalytic domain to illustrate active site architecture with ligand represented in balls and sticks. The ligand of PDB-ID: 2YLA was manually docked in HEX-2 and EndoE.

As the enzymatic activity experiments indicated that *T. suis* HEX-2 removes GalNAc and GlcNAc residues from glycan substrates, its newly resolved catalytic domain was superimposed on that of *S. pneumoniae* GH20C (Supplementary Figure S6), which has been cocrystallized with the GalNAc and GlcNAc reaction products, which are also surrogates for actual substrates. It is noted that those monosaccharides are distorted in the catalytic site. 265 residues out of 320 aligned with a rmsd of 1.9 Å and major differences are observed at the level of surface loops in particular those surrounding the active site pocket and in particular the −1 subsite as described below. This structural analysis indicated that either monosaccharide is capable of effectively binding to the predicted −1 subsite (Figure 7A), whereby the key contacts will be conserved with the 1-hydroxyl and 2-acetamido groups. Based on the presented structure, a number of residues are predicted to participate in substrate binding within the structure (Table 2, Figure 7). These include the Asp198 and Glu199 of the His/Asn-Xaa-Gly-Xaa-Asp-Glu motif shared with other GH20 hexosaminidases (i.e., residues 282 and 283 of the full theoretical sequence), whereby the Asp and Glu are the polarizing and general acid/base residues as demonstrated by studies on members of this retaining enzyme family, including a photoaffinity labeling study on human hexosaminidase B.^41^ Four aromatic groups forming the bottom (Trp348) and the walls of the active site pocket around the acetamido group (Trp246, Trp272, and Tyr274) are also conserved (Figure 7). There are, though, differences in terms of contacts with the monosaccharide for hydroxyls at the 3, 4, and 6 positions and in the binding side topology around the hydroxymethyl group resulting from differences in several surface loops between HEX-2 and GH20C (Figure 7). The loop containing Arg94 in GH20C is a little shorter in HEX-2, but the side chain nitrogen of Lys66 occupies the same position as the NH1 atom of Arg94 and therefore could interact with the 3-hydroxyl but also with the 4-hydroxyl in GlcNAc. In GH20C, the 4 and 6-hydroxyls interact with the carboxylate atoms of Asp375 while the hydroxymethyl conformation is blocked by hydrophobic interactions with Trp339 and Tyr309 (Figure 7B). In HEX-2, those residues are replaced by the side chains of Tyr351, Gly305, and Ala275, respectively. The sequence of the surface loops containing Gly305 and Tyr351 are nonconserved leading to a very different conformation, which, along with the replacement of Tyr309 by an alanine, results in altered interactions and a more open active site pocket in HEX-2. In order to avoid steric clashes with Tyr351, the hydroxymethyl has to rotate to stack along the aromatic ring and in that orientation the 6-hydroxyl group would make an H-bond with the main chain oxygen of Gly305 (Figure 7A); thus, it can be expected that conformational changes upon substrate binding would optimize the interactions and result in ordering of the missing amino acids 200–220. The differences in sequence and hence in conformation of six surface loops surrounding the −1 subsite have a strong impact in the overall architecture of the active site and in the formation of additional subsite (in HEX-2:65–74, 195–206, 247–254, 273–285, 305–315, 348–360). In HEX-2, the loop containing Lys66 corresponding to the one containing Arg94 in GH20C, will prohibit the formation of a −2 subsite as found in the *Bifidobacterium bifidum* lacto-*N*-biosidase LNBase (Figure 7D–F).^42^ The other five loops create two grooves that could accommodate at least +1 and +2 subsites and a branched glycan (Figure 7D). The X groove is also found in other hexosaminidases such as in the endoglycosidase E GH20 from *Enterococcus faecalis*, but is shallower in HEX-2 (Figures 7D,F).^43^

**Table 2:** List of Residues Potentially Involved in the Binding of Substrates as Compared to *S. pneumoniae* GH20C β-Hexosaminidase^a^

substrate binding	*T. suis* HEX-2	*S. pneumoniae*β-hexosaminidase
GalNAc/GlcNAc H-bond O3	K66	R94
GalNAc/GlcNAc H-bond N2	D198	D222
GalNAc/GlcNAc H-bond O1	E199	E223
GalNAc/GlcNAc hydrophobic N-Acetyl	W246	W266
GalNAc/GlcNAc hydrophobic N-acetyl	W272	W306
GalNAc/GlcNAc O7	Y274	Y308
GalNAc/GlcNAc hydrophobic ring	W348	W373
GalNAc O6/GlcNAc O4 & O6	G305	D375
GalNAc/GlcNAc hydrophobic O6	Y351	

a Residue numbering is for the crystallized forms.

## Discussion

The phylogenetic analysis of eukaryotic GH20 hexosaminidases presented here has provided new insights into the origin of these enzymes in nematodes (Figures 1 and 2). As indicated by previous studies, it was confirmed that the aforementioned HEXD is the most similar human homologue to the subfamily 1 hexosaminidases present in nematodes.^26^ Furthermore, the phylogenetic reconstruction revealed that the four major branches of subfamily 1 hexosaminidases found in nematodes derive from a single ancestor, indicating that the genes evolved through numerous duplications and later speciation. While most nematodes analyzed possess multiple predicted subfamily 1 GH20 hexosaminidases, the examined clade I species (Dorylaimia; *Soboliphyme baturini*, *Trichuris* spp., and *Trichinella* spp.) have apparently only one such sequence forming a separate GH20 subbranch (Figure 2).

Previously published studies have suggested that subfamily 1 GH20 enzymes prefer aryl β-*N*-acetylgalactosaminides over β-*N*-acetylglucosaminides and are generally more efficiently inhibited by galacto-epimers of hexosaminidase inhibitors as compared to those in the gluco-configuration^2,7,39^ with our unpublished data on *C. elegans* HEX-2 also indicating a reduction in *K*_i_ for Gal-PUGNAc as opposed to PUGNAc (500-fold). In the case of *T. suis* HEX-2, low activity toward pNP-β-GlcNAc was detected, but it had a high activity with pNP-β-GalNAc and GalNAc-isofagomine was the most effective inhibitor of those tested (Figure 3), demonstrating its close relationship with other subfamily 1 enzymes.

The situation with N-glycan substrates is more complex than for the simple aryl glycosides: while only the “lower” GlcNAc was removed from biantennary glycans with nonreducing terminal GlcNAc residues, all terminal GalNAc residues and one subterminal GlcNAc was lost from biantennary glycans with LacdiNAc motifs (Figures 4 and 5). This is akin to the activity of *C. elegans* HEX-2; thus, the phylogenetic designation of the *T. suis* enzyme as an HEX-2 matches its enzymatic activity. On the other hand, *C. elegans* HEX-3 only removes the “lower” GlcNAc, but *C. elegans* HEX-4 is completely GalNAc-specific. Thus, despite the galacto-epimer bias for the simple substrates and inhibitors as for other subfamily 1 enzymes, *T. suis* HEX-2 can also digest a specific GlcNAc-containing linkage at slightly acidic pH in the same manner as insect FDL enzymes, which are members of GH20 subfamily 2. In contrast, plant hexosaminidases, which remove nonreducing terminal GlcNAc, can remove both such residues from N-glycans.^2,12^

Overall, we assume that *T. suis* HEX-2 has a role in the biosynthesis of the major paucimannosidic N-glycans known to occur in this parasite.^24^ The subtlety of its specificity toward LacdiNAc-type substrates are of interest: while *C. elegans* HEX-4 can remove GalNAc from fucosylated and phosphorylcholine-modified LacdiNAc-type motifs,^46^ the presence of an antennal fucose appears to block the action of *T. suis* HEX-2. Unlike *C. elegans*, the *T. suis* N-glycome is rich in antennal fucose motifs but lacks the phosphorylcholine-modified chito-oligomer modifications found in a variety of other nematodes; also *C. elegans* HEX-4 appears to have a role in N-glycan biosynthesis in the Golgi apparatus and ablation of its gene leads to an increase in GalNAc-containing N-glycans in the model nematode.^21^ Thus, the fine biosynthetic control mechanism involving LacdiNAc-containing glycans may be lacking in *T. suis* and so may be a partial explanation for its species-specific glycome.^47^ On the other hand, the catabolism of GalNAc- and GlcNAc-containing N-glycans at acidic pH may be performed in *T. suis* by HEX-1, which belongs to GH20 subfamily 2; at least, the potentially related *T. spiralis* enzyme can degrade such structures.^22^

The GH20C enzyme from *Streptococcus pneumoniae*,^39^ which is the closest related protein (29% identity) with a crystal structure, has some bias toward GalNAc, but far less than *T. suis* HEX-2. Nevertheless, as GH20C was also cocrystallized with GalNAc, GlcNAc, and some inhibitors, the superimposition with its structure is the most meaningful possible approximation; these structures were the basis for predicting potential interactions of *T. suis* HEX-2 with GalNAc and GlcNAc in subsite −1 (Figure 6B). The two enzymes only superposed well in the catalytic domain (Figure 7A). Although the modeled conformations of the two monosaccharides are subtly different, H-bond interactions of the anomeric hydroxyl group with Glu199 (corresponding to Glu223 of GH20C) and the 2-acetamido group with Asp198 and Tyr274 (corresponding to Asp222 and Tyr308 of GH20C) would be conserved as well as the hydrophobic or stacking interactions with aromatic rings of Trp246, Trp 272, and Trp348 (corresponding to Trp residues 266, 306, and 373 of GH20C). The cocrystallization with GH20C indicates a different H-bonding pattern of the 4-hydroxyl groups of GalNAc and GlcNAc to either guanidino amino group of Arg 94 (Figure 7B), enabling binding of both monosaccharides; the corresponding Lys66 in HEX-2 only has a single side chain amino group, which may affect relative specificities for the two pNP-substrates as well as the inhibitors. Although the *in silico* prediction using AlphaFold was close to the model from the crystal structure (Figure 7C and Supplementary Figure S5), some deviations were found, thereby highlighting the value of an experimentally based approach. The presence of a Zn^2+^ ion near the proposed active site was not expected, its role is unknown but this is found in some other glycosidases, including Golgi mannosidase II.^48^ Another example monosaccharide-releasing hexosaminidase with a high GalNAc-bias is the recently crystallized *Paenobacillus* TS12 NgaP2 (PDB 8K2L);^49^ despite overall structural superposition of their catalytic regions being possible, a meaningful comparison regarding side chains determining substrate specificity is difficult as NgaP2, assigned to the GH123 family, has only 11% identity with HEX-2.

Few comparisons can be made to eukaryotic GH20 hexosaminidases as the four proteins for which there are crystallographic data are all of subfamily 2. Nevertheless, the key role of an Asp-Glu pair and a Tyr residue up to 100 amino acids toward the C-terminus in binding GalNAc, GlcNAc, or inhibitors is conserved.^50−52^ However, the general architecture of subfamily 2 enzymes contrasts with that of *T. suis* HEX-2, whereby the active site is closer to the N-terminus in subfamily 1. As most GH20 crystallographic studies are of bacterial enzymes, the crystal structure described here is particularly valuable, as it is the first eukaryotic one from this subfamily. Thus, this structure and data presented here coupled with further studies will allow for a better understanding of the substrate specificities of invertebrate hexosaminidases and how they have evolved.

## Experimental Procedures

### Phylogenetic Analyses

#### Eukaryotic Tree

To find all eukaryotic sequences, which belong to the GH20 hexosaminidase family, the Enzyme Function Initiative-Enzyme Similarity Tool (EFI-EST) was used.^53^ Two protein families IPR015883 (subfamily 1) and IPR025705 (subfamily 2) were found, and all eukaryotic data was downloaded and stored on a local disk. The data has been processed to decrease the number of input sequences; any sequences below 300 or over 750 amino acids were removed. Next, sequences were used to build an alignment with MAFFT^54^ and then the TrimAl tool was used for alignment trimming.^55^ Thereafter, the data was used as input to calculate a new phylogeny tree using the FastTree tool^56^ on the local computer and visualized with iTOL.^57^

#### Nematode Tree

Characterized GH20 sequences from *C. elegans* were taken from the Wormbase database (gene names, HEX-1 – CE07499; HEX-2 – CE36785; HEX-3 – CE41720; HEX-4 – CE46668; HEX-5 – CE53609) and used as a query the whole Nematode proteome (NCBI 11.01.2023) using the hidden Markov Models algorithm from phmmer. All found sequences were used to build an alignment with MAFFT^54^ and subsequently the final approximately maximum-likelihood phylogenetic tree was built with IQ-tree.^58^ The resulting phylogeny tree was limited to one homologue per species and visualized with iTOL.^57^

### Cloning and Purification

The *T. suis* HEX-2 open reading frame sequence, excluding the region encoding the cytosolic, transmembrane, and stem domains (i.e., residues 85–620 of the predicted protein), was synthesized by GenScript, based on the sequence with NCBI database ID KFD67184. The hexosaminidase sequence was cloned into the pPICZαA plasmid (Invitrogen) without the native stop codon using the Gibson Assembly Cloning Kit (primers: Forward/Long: 5′-AGAGAGGCTGAAGCTGAATTCACGATGAAAGTGTATCGATGGCGA-3′, Forward/Short: 5′-AGAGAGGCTGAAGCTGAATTCACGGTGTTTATTCCGAAACGT-3′, Reverse: 5′-GACGGCACGCGTCGTATCGATAG-3′). Ligation products were transformed into 5-alpha competent *Escherichia coli* (New England Biolabs, C2987) prior to selection on zeocin. The sequenced expression vectors were linearized and transformed into *P. pastoris* (GS115 strain), and colonies were selected on Zeocin and expression performed with methanol induction at 30 °C as previously described.^12^

Purification of the recombinant proteins from the culture media was performed with an ÄKTA go protein purification system with HisTrap High Performance 1 mL column (Cytiva); samples were applied in binding buffer (25 mM sodium phosphate, 150 mM NaCl, pH 7.4), and the column was washed before using a gradient of elution buffer (25 mM sodium phosphate, 150 mM NaCl, 500 mM imidazole, pH 7.4). After purification, eluted fractions were tested for purity by SDS-PAGE; fractions of interest were pooled, concentrated using an Amicon Ultra-0.5, Ultracel-30 Membrane with a 30 kDa cutoff (Merck Millipore), and exchanged into storage buffer (20 mM Tris-HCl, 150 mM NaCl, pH 7.5). His-tagged forms of the hexosaminidases were detectable after western blotting using the anti-His monoclonal antibody (1:10000; Sigma-Aldrich) and alkaline-phosphatase conjugated antimouse IgG (1:10000; Sigma-Aldrich). The resulting secreted long (536 residues corresponding to residues 85–620 of the predicted protein) and short (residues 135–620 of the predicted protein, i.e., 51–536 encoded by the construct) forms both had a C-terminal His-tag. An alternative long form with an N-terminal His/FLAG-tag was also expressed (Supplementary Figure S4A) and was active, but lost the N-terminal tag due to proteolysis prior to purification. To examine the monomeric or multimeric status of HEX-2 (Supplementary Figure S4B), native gel electrophoresis was performed as for SDS-PAGE, except for exclusion of SDS and reducing agents from the sample buffer and gel; the resulting gel was fixed in a solution of 40% (v/v) ethanol and 10% (v/v) acetic acid, incubated with 0.125% (w/v) glutaraldehyde, 0.2% (w/v) sodium thiosulfate, 6.8% (w/v) sodium acetate in 30% (v/v) ethanol, and then with 0.25% (w/v) silver nitrate and 0.015% (v/v) formaldehyde prior to development overnight in 2.5% (w/v) sodium carbonate and 0.0075% (v/v) formalehyde.

### Hexosaminidase Assays

The standard enzyme activity test was performed in 96-well plates. Typically, a mixture of 2.5 μL of pNP-β-GalNAc (100 mM in dimethyl sulfoxide), 46.5 μL of McIlvaine buffer pH 6.5,^59^ and 1 μL of enzyme was incubated for 1 h at 37 °C; 200 μL of stop solution (0.4 M glycine/NaOH, pH 10.4) was added and the absorbance measured with an Infinite 200 PRO instrument (Tecan). Inhibitors were prepared as previously reported.^30,34,60−63^ For tests with remodelled glycopeptides^12^ or 2D-HPLC fractions,^35,47^ a 1 μL aliquot was mixed with 0.2 μL enzyme and 0.8 μL of 50 mM ammonium acetate solution, pH 6.5. After overnight incubation at 37 °C, 0.5 μL of the mixture was analyzed by MALDI-TOF-MS (Autoflex Speed, Bruker, Bremen) with 6-aza-2-thiothymine (ATT) as the matrix; data were analyzed with the Flexanalysis (Bruker) program.

### X-ray Crystallography

Initial protein crystallization screening was performed using the robotized HTXlab platform (EMBL, Grenoble, France) in a sitting drop vapor diffusion setup by mixing 100 nL of protein solution (5.1 mg/mL) and 100 nL of crystallization solution prior storage at 20 °C in a visible and UV Imaging Robot. A second screening was performed using different commercially available crystallization screens at CERMAV in a hanging drop vapor diffusion setup by mixing 1 μL of protein solution (6 mg/mL) and 1 μL of crystallization solution. The screening plate was kept in a vibration-free incubator (Molecular Dimensions, Calibre Scientific, Rotherham, UK) at 19 °C. Crystal clusters were obtained from condition 18 of the Clear Strategy Screen II (Molecular dimensions) consisting of 20% PEG 1500, 0.15 M potassium thiocyanate, and 0.1 M Tris pH 7.5. 15% PEG 1000 were added to the mother liquor as a cryoprotectant prior to mounting a single crystal in a cryoloop (Molecular Dimensions) and flash freezing in liquid nitrogen. The diffraction data were collected at Synchrotron SOLEIL, Beamlines, Proxima-1 (Saint-Aubin, France) using an Eiger 16 M detector (Table 1). The XDS^64^ and XDSme^65^ were used to process the data and further steps were performed with CCP4, version 8.25–27.^66,67^ As the crystal diffracted anisotropically, data was processed using the STARANISO server and the aimless CCP4 program. The structure of HEX-2 was solved by molecular replacement where AlphaFold^68^ was used to generate a search model for PHASER.^69^ Iterated maximum likelihood refinement and manual building of the resulting electron density maps were respectively performed using REFMAC 5.8^70^ and Coot.^71^ Five percent of the reflections were used for cross-validation analysis, and the behavior of *R*_free_ was employed to monitor the refinement strategy. Water molecules were added by using Coot and subsequently manually inspected.

## Data Availability

Data described in the manuscript are shown in the figures; the coordinates of the *T. suis* HEX-2 crystal structure were in the Protein Data Bank (PDB) under code 8QK1.^72^

## Supporting Information Available

full phylogeny of nematode hexosaminidases; predicted sequence of *T. suis* HEX-2 as well as recombinant forms and alignments of *T. suis* HEX-2 with *C. elegans* HEX-2 and other hexosaminidases; further data on the recombinant *T. suis* HEX-2; further views of the X-ray crystal data (PDF)

## Accession Codes

HEX-2 from *Trichuris suis* has the NCBI protein accession number KFD67184.

## ABBREVIATIONS

HEX: hexosaminidase
PC: phosphorylcholine
pNP: p-nitrophenyl
