# Supplementary material for: Bioinformatic, Enzymatic, and Structural Characterization of Trichuris suis Hexosaminidase HEX-2
Source: Biochemistry. 2024 Jul 26;63(15):1941–54. doi: 10.1021/acs.biochem.4c00187 (PMC11308363; doi:10.1021/acs.biochem.4c00187)
Supplement: Supplementary file 1 — bi4c00187_si_001.pdf [file bi4c00187_si_001.pdf]

Bioinformatic, enzymatic and structural characterization of *Trichuris suis* hexosaminidase HEX-2

Zuzanna Dutkiewicz[1,†], Annabelle Varrot[2], Karen J. Breese[3], Keith A. Stubbs[3,4], Lena Nuschy[1], Isabella Adduci[5], Katharina Paschinger[1] and Iain B. H. Wilson[1,]*

[1] Institut für Biochemie, Department für Chemie, Universität für Bodenkultur, Muthgasse 18, 1190 Wien, Austria

[2] Univ. Grenoble Alpes, CNRS, CERMAV, 38000 Grenoble, France

[3] School of Molecular Sciences, University of Western Australia, Crawley, WA 6009, Australia

[4] ARC Training Centre for Next-Gen Technologies in Biomedical Analysis, School of Molecular Sciences, University of Western Australia, Crawley, WA 6009, Australia

[5] Institut für Parasitologie, Department für Pathobiologie, Veterinärmedizinische Universität Wien, Veterinärplatz 1, A-1210 Wien, Austria

† Current address: Institut für Mikrobiologie, Universität Innsbruck, A-6020 Innsbruck, Austria

* Correspondence to: iain.wilson@boku.ac.at

Supporting Information

Supplementary Figures S1-S6

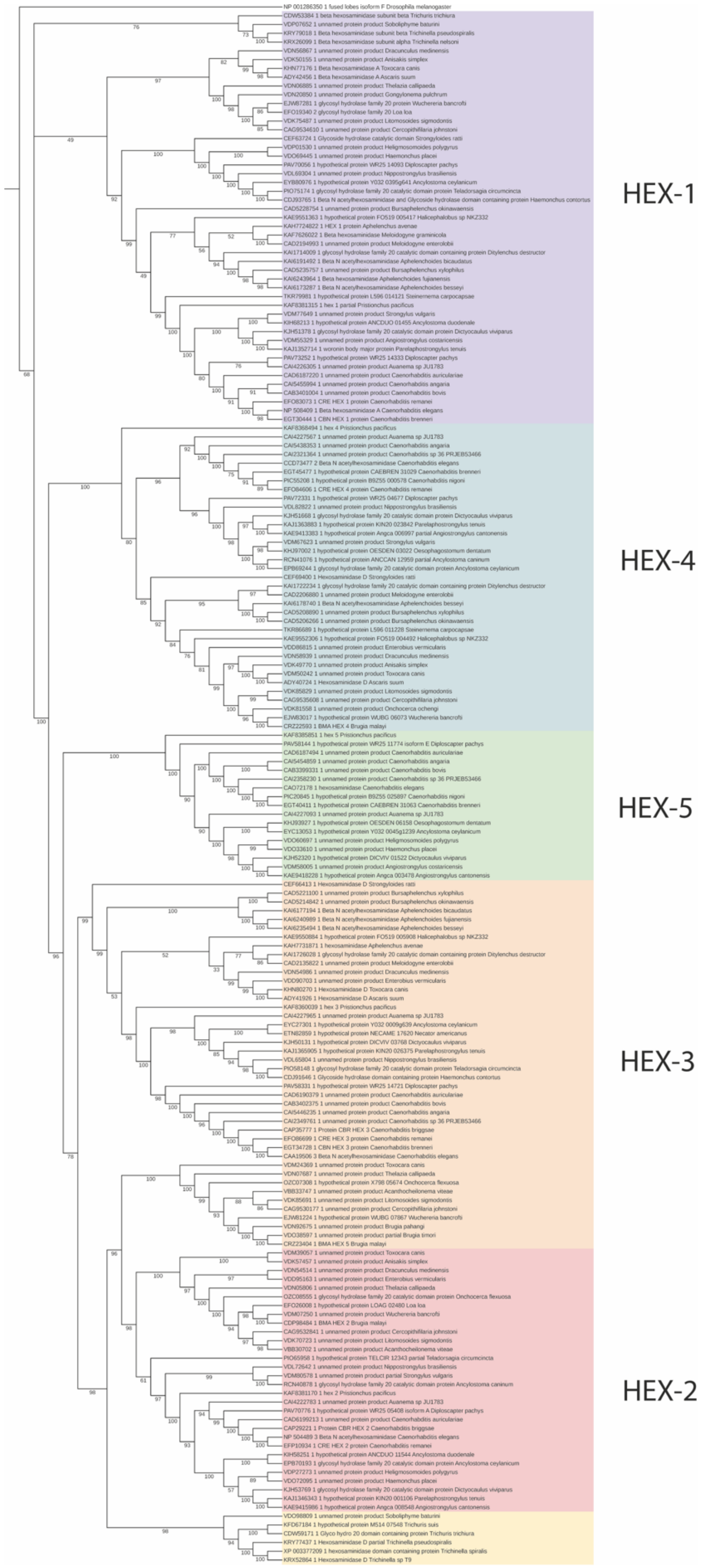

Supplementary Figure S1A: Full phylogenetic tree of nematode subfamily 1 GH20 hexosaminidases – these data underly ***Figure 2*** in the main text. *D. melanogaster* FDL (fused lobes hexosaminidase) was used as an out group. Bootstrap values of more than 70 are shown.

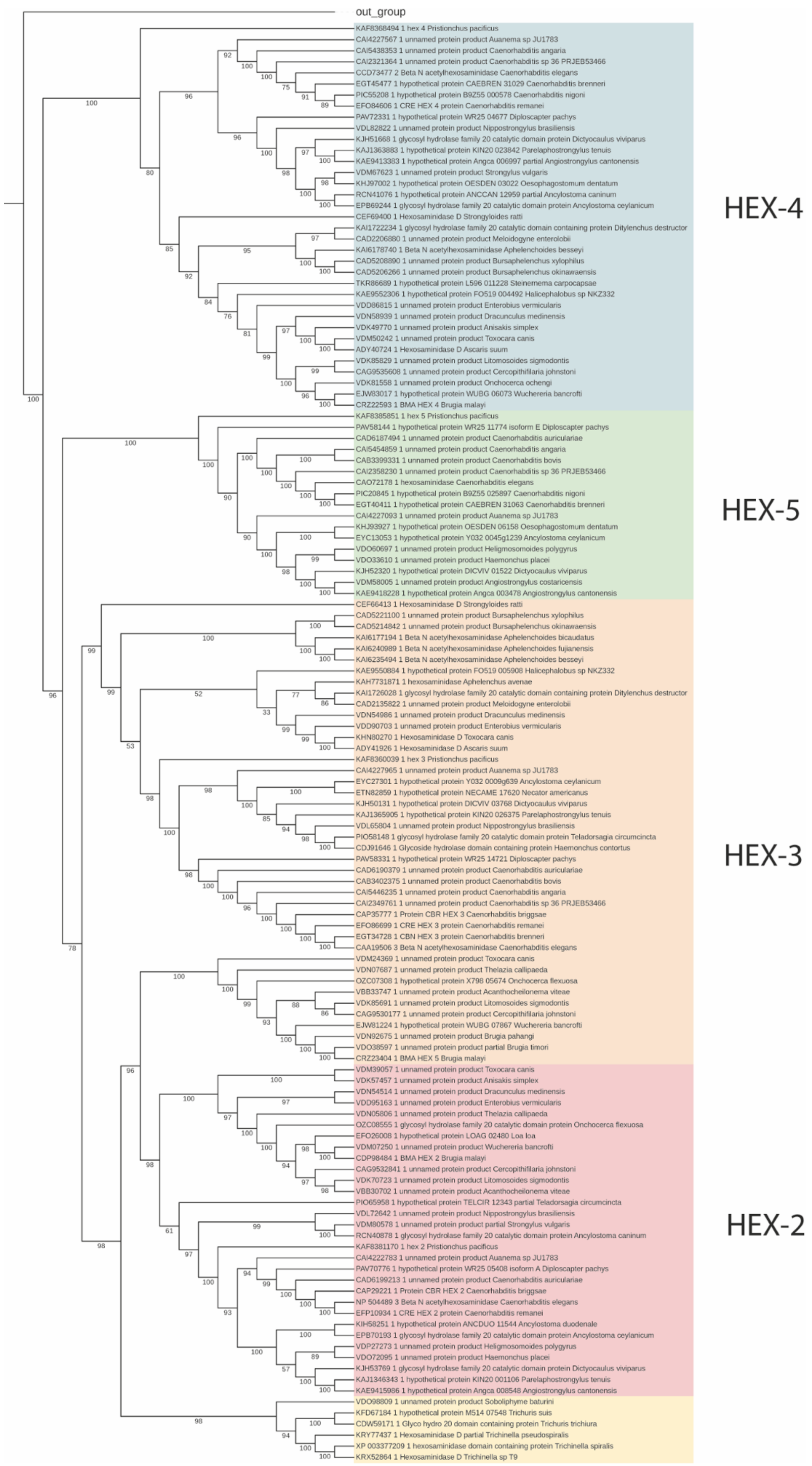

Supplementary Figure S1B: Full phylogenetic tree of all nematode GH20 hexosaminidases – these data underly ***Figure 2*** in the main text. *D. melanogaster* FDL was used as an out group. Bootstrap values of more than 70 are shown.

A) Full theoretical sequence:

```
  1 MTGKKCAFVQ HKYSKLRQDY VLQDDNEVAT GRSVADSNVR RYFTVMKVYR WRGKPAFAFI TVLTVILLII GYHTITSRHG
 81 DTVIHEGVFQ RGAAMRKRTV YGQVDEKSSP TSTSTDLTMK TTTTYSQNLK EPPPSNVFIP KRRIVHLDLK GAAPKPQHFR
161 AFFEYFVRIG ATGILIEWED MFPYEGRLSD LRNGDAYSAD DVRMILSTAD QLRLEVIPLV QTIGHLEWLL KTHKFYSFRE
241 NPRNPQSVCV SNAEAVDLVL HLVDQVMAFH KDYGQFVHIG ADEVYQYGEC SRCVARMNKE NLRREDLLLR HIVNVSKHVK
321 TKYGKNVLMW HDMIANIDAS LAEKYDLKNL VEPVLWNYAE DLEAFLPMGI WETFSAMVPY MWGSSAFKGA DSPTRYHSNV
401 KHYLENHISW IKQMSTASEK FREFRGLIFT GWQRYDHFAV LCEFLPIGIP SLTVNMLTIR NGRFDASVND QAISIMQCVT
481 GSDVKGDLYG CRFPGSDIYH HVQLLHEKKG EIEKLLLQQS VQGWLSNIAI DYNMSSPWYM NLIVPDLMTY KNQMIELSLN
561 IRQAMLEMFY ENAVDEFLFT YVDPVINHLQ RLLDRATAIQ RRDEFPVRPF PIKRTIDTTR
```

B) Long secreted form:

```
       HEGVFQ RGAAMRKRTV YGQVDEKSSP TSTSTDLTMK TTTTYSQNLK EPPPSNVFIP KRRIVHLDLK GAAPKPQHFR
161 AFFEYFVRIG ATGILIEWED MFPYEGRLSD LRNGDAYSAD DVRMILSTAD QLRLEVIPLV QTIGHLEWLL KTHKFYSFRE
241 NPRNPQSVCV SNAEAVDLVL HLVDQVMAFH KDYGQFVHIG ADEVYQYGEC SRCVARMNKE NLRREDLLLR HIVNVSKHVK
321 TKYGKNVLMW HDMIANIDAS LAEKYDLKNL VEPVLWNYAE DLEAFLPMGI WETFSAMVPY MWGSSAFKGA DSPTRYHSNV
401 KHYLENHISW IKQMSTASEK FREFRGLIFT GWQRYDHFAV LCEFLPIGIP SLTVNMLTIR NGRFDASVND QAISIMQCVT
481 GSDVKGDLYG CRFPGSDIYH HVQLLHEKKG EIEKLLLQQS VQGWLSNIAI DYNMSSPWYM NLIVPDLMTY KNQMIELSLN
561 IRQAMLEMFY ENAVDEFLFT YVDPVINHLQ RLLDRATAIQ RRDEFPVRPF PIKRTIDTTR AVDHHHHHH
```

C) Alignment with C. elegans HEX-2

```
Query  124  TYSQNLKEPPPSNVFIPKRRIVHLDLKGAAPKPQHFRAFFEYFVRIGATGILIEWEDMFP  183
            T    ++ P   N F  K  ++H DLKGA PK  +F        + GATGIL+EWEDMFP
Sbjct  96   TQEVKIERPSRDNEFY-KNVVIHFDLKGAPPKVDYFLDLLRLIAKGGATGILLEWEDMFP  154

Query  184  YEGRLSDLRNGDAYSADDVRMILSTADQLRLEVIPLVQTIGHLEWLLKTHKFYSFRENPR  243
            + G+L   +N DAYS  DV MILS A +L+L+VIPLVQT GHLEW+LK  +   +REN
Sbjct  155  WTGKLEQFKNTDAYSESDVDMILSEATKLKLDVIPLVQTFGHLEWILKYEEMRKYRENDA  214

Query  244  NPQSVCVSNAEAVDLVLHLVDQVMAFHKDYG-QFVHIGADEVYQYGECSRCV--ARMNKE  300
             PQ +C+ N E V+ V  ++ QV   H  YG  F HIGADE +++G C   +   + N +
Sbjct  215  YPQVLCLGNEEGVEFVREMIRQVAKKHAKYGIPFFHIGADEAFEFGVCQESLDWIKKNGK  274

Query  301  NLRREDLLLRHIVNVSKHVKTKYGKN--VLMWHDMIANIDASLAEKYDLKNLVEPVLWNY  358
            N R++ L L H+  +++  K + G +  +L WHDM+ + D+ L +  +L  +++PV+W+Y
Sbjct  275  NGRKQLLALAHLKAIAEFAKQQTGDSTQILAWHDMLKDFDSRLIKNLELGQIIQPVVWDY  334

Query  359  AEDLEAFLPMGIWETFSAMVPYMWGSSAFKGADSPTRYHSNVKHYLENHISWIKQMSTAS  418
            +E++   L   I+   +   P MW SSA+KGA+ P+   S V+HY  N+ +WI+
Sbjct  335  SENI-ITLNDYIFSALAENFPTMWASSAYKGANYPSASTSEVRHYETNNRNWIRTKQNQE  393

Query  419  EKFRE-FRGLIFTGWQRYDHFAVLCEFLPIGIPSLTVNMLTIRNG-RFD-ASVNDQAISI  475
             KF+  F+G+I TGWQRYDH A LCE LPIG  S+ + M    N    D      +A ++
Sbjct  394  RKFKNGFQGIIVTGWQRYDHLAGLCETLPIGTASMMLQMQIALNAPALDLEGTRQKAATL  453

Query  476  MQC----VTGSDVKGDLYGCRFPGSDIYHHVQ-----LLHEKKGEIEKLLLQQSVQGWLS  526
            ++C    V G  V  +   C++ G   Y   Q     L      E+ K      + GW +
Sbjct  454  LECQGFNVDGVKVVSN--QCKYRGFQTYLIYQSEVPNLFARIDSELSK---NHHLMGWAN  508

Query  527  NIAIDYNMSSPWYMNLIVPDLMTYKNQMIELSLNIRQAMLEMFYENAVDEFLFTYVDPVI  586
                 YN+S  WY   ++P +     Q   +  ++R +M ++++EN +DEF++  +  +
Sbjct  509  RYNRKYNISQNWYHREMLPFVQQLVGQYDRVESDLRASMKDLYFENTIDEFIYENLGEMS  568

Query  587  NHLQRLLDRATAIQRRDEFPVRPFPIKR  614
              L   L+    + +   +P R FPIK+
Sbjct  569  EKLHGYLEEIQRLDKLRAWPKRHFPIKK  596
```

Supplementary Figure S2: (A) Sequence of the predicted subfamily 1 hexosaminidase (HEX-2) from *T. suis* (hypothetical protein M514_07548; NCBI accession KFD67184). The predicted transmembrane domain absent from all recombinant protein constructs is in red, the potential N-glycosylation sites are underlined and the conserved HIGADE active site region is indicated in bold; the actual initial methionine residue is unknown but two are N-terminal to the transmembrane domain. (B) Sequence of the recombinant long secreted form (expected M_r 63 kDa) used for X-ray crystallography; the N-terminal disordered region absent from the recombinant short secreted form (expected M_r 57 kDa) is italicized. (C) Alignment of *T. suis* and *C. elegans* HEX-2 (36% identity over 508 residues).

```
           41        50        60        70        80        90        100
TsHEX-2   YSQNLKEPPPSNVFIPKRRIVHLDLKGAAPKPQHFRAFFEYFVRIGATGILIEWEDMFPY
CeHEX-2   QEVKIERPSRDNEFY.KNVVIHFDLKGAPPKVDYFLDLLRLIAKGGATGILLEWEDMFPW
Hs_HexA   ....PRFPHRGLLLDTSRHYLPLSSILDTLDVMAYNKLNVFHWHLVDDPSFPYESFTFPE
Hs_HexB   ....PRFSHRGILIDTSRHYLPVKIILKTLDAMAFNKFNVLHWHIVDDQSFPYQSITFPE
Dm_fdl    ....PKFRYRGLMLDTSRHFFSVESIKRTIVGMGLAKMNRFHWHLTDAQSFPYISRYYPE
OfHex1    ....PVYPYRGILLDTARNYYSIESIKRTIEAMAAVKLNTFHWHITDSQSFPFVTTKRPN

                110       120       130       140
TsHEX-2   EGRLSDLRN.GDAYSADDVRMILSTADQLRLEVIPLVQTIGH..LEW........LLKTH
CeHEX-2   TGKLEQFKN.TDAYSESDVDMILSEATKLKLDVIPLVQTFGH..LEW........ILKYE
Hs_HexA   LMRKGSYNPVTHIYTAQDVKEVIEYARLRGIRVLAEFDTPGHT.LSWGPGI...PGLLTP
Hs_HexB   LSNKGSYS.LSHVYTPNDVRMVIEYARLRGIRVLPEFDTPGHT.LSWGKGQ...KDLLTP
Dm_fdl    LAVHGAYSE.SETYSEQDVREVAEFAKIYGVQVIPEIDAPAHAGNGWDWGPKRGMGELAM
OfHex1    LYKFGALSP.QKVYTKAAIREVVRFGLERGVRVLPEFDAPAHVGEGWQ......DTDLTV

         150       160       170       180       190       200
TsHEX-2   KFYSFRENPRNPQSVCVSNAEAVDLVLHLVDQVMAFHKDYG.Q..FVHIGADEVYQ..YG
CeHEX-2   EMRKYRENDAYPQVLCLGNEEGVEFVREMIRQVAKKHAKYGIP..FFHIGADEAFE..FG
Hs_HexA   CYSGSEPSG.....TFGPVNPSLNNTYEFMSTFFLEVSSVF.PDFYLHLGGDEVDFTCWK
Hs_HexB   CYSRQNKLD.....SFGPINPTLNTTYSFLTTFFKEISEVF.PDQFIHLGGDEVEFKCWE
Dm_fdl    CINQQPWSFYCGEPPCGQLNPKNNYTYLILQRIYEELLQHTGPTDFFHLGGDEVNLDCW.
OfHex1    CFKAEPWKSYCVEPPCGQLNPTKDELYQYLEDIYSDMAEVFDTTDIFHMGGDEVSEACWN
                                                       catalytic site

                 210       220       230       240       250
TsHEX-2   ECSRCV..ARMNKENLRREDLLL..RHIVNVSKHVKTKYGKN...VLMWHDMIANIDASL
CeHEX-2   VCQESLDWIKKNGKNGRKQLLAL..AHLKAIAEFAKQQTGDSTHQILAWHDMLKDFDSRL
Hs_HexA   SNPEIQDFMRKKGFG...EDFKQLESFYIQTLLDIVSSYG...KGYVVWQEVFDNK....
Hs_HexB   SNPKIQDFMRQKGFG...TDFKKLESFYIQKVLDIIATIN...KGSIVWQEVFDDK....
Dm_fdl    .AQYFNDTDLRGLWC....DFM.....LQAMARLKLANNGVAPKHVAVWSSALTNTK...
OfHex1    SSDSIQNFMMQNRWDLDKESFLKLWNYFQQKAQDKAYKAFGKKLPLILWTSTLTNYKHID

            260       270       280       290       300       310
TsHEX-2   AEKYDLKNLVEPVLWNYAEDLEAFLPMGIWETFSAMVPYMWGSSAFKGADSPTRYHSNVK
CeHEX-2   IKNLELGQIIQPVVWDYSENIIT.LNDYIFSALAENFPTMWASSAYKGANYPSASTSEVR
Hs_HexA   .VKIQPDTIIQVWREDIPVNYMKELE...LVTKAGFRALLSAPWYLNRISYGPDWKDFYV
Hs_HexB   .AKLAPGTIVEVWKDSA...YPEELS...RVTASGFPVILSAPWYLDLISYGQDWRKYYK
Dm_fdl    .CLPNSQFTVQVWGGSTWQENYDLLDNGYNVIFSHVDAWYLDCGFGSWRATGDAACAPYR
OfHex1    DYLNKDDYIIQVWTTGVDPQIKGLLEKGYRLIMSNYDALYFDCGYGAWVGAGNNWCSPYI

            320       330       340       350       360       370
TsHEX-2   HYLENHISWI..KQMSTASEKREFRGLIFTGWQRYDHFAVLCEFL.PIGIPSLTVNMLTI
CeHEX-2   HYETNNRNWIRTKQNQERKFKNGFQGIIVTGWQRYDHLAGLCETL.PIGTASMMLQMQIA
Hs_HexA   VEPLAFEGTP........EQKALVIGGEACMWGEYVDNTNLVPRLWPRAGAVAERLWSNK
Hs_HexB   VEPLDFGGTQ........KQKQLFIGGEACLWGEYVDATNLTPRLWPRASAVGERLWSSK
Dm_fdl    TWQNVYKHRPWERMRLDKKRKKQVLGGEVCMWTEQVDENQLDNRLWPRTAALAERLWTDP
OfHex1    GWQKVYDNSP...AVIALEHRDQVLGGEAALWSEQSDTSTLDGRLWPRAAALAERLWAEP
```

Supplementary Figure S3: Alignment of *T. suis* and *C. elegans* HEX-2 (both subfamily 1), human HEXA and HEXB, *Drosophila melanogaster* FDL and *Ostrinia furnacalis* OfHex1 (subfamily 2). Sequences were shortened to the region of highest homology and aligned using Multalin, http://multalin.toulouse.inra.fr/ - the file was processed on https://espript.ibcp.fr/ESPript/ESPript/ Crystal structures exist for human hexosaminidases A and B as well as OfHex1, while HEX-2 and FDL specifically remove the β1,2GlcNAc from the α1,3-mannose of N-glycans. Only the catalytic site as well as a region towards the N-terminus is well conserved between the two subfamilies. Highlighted in blue are selected key subfamily-specific residues identified in this and other structural studies (other than those conserved across the families in red).

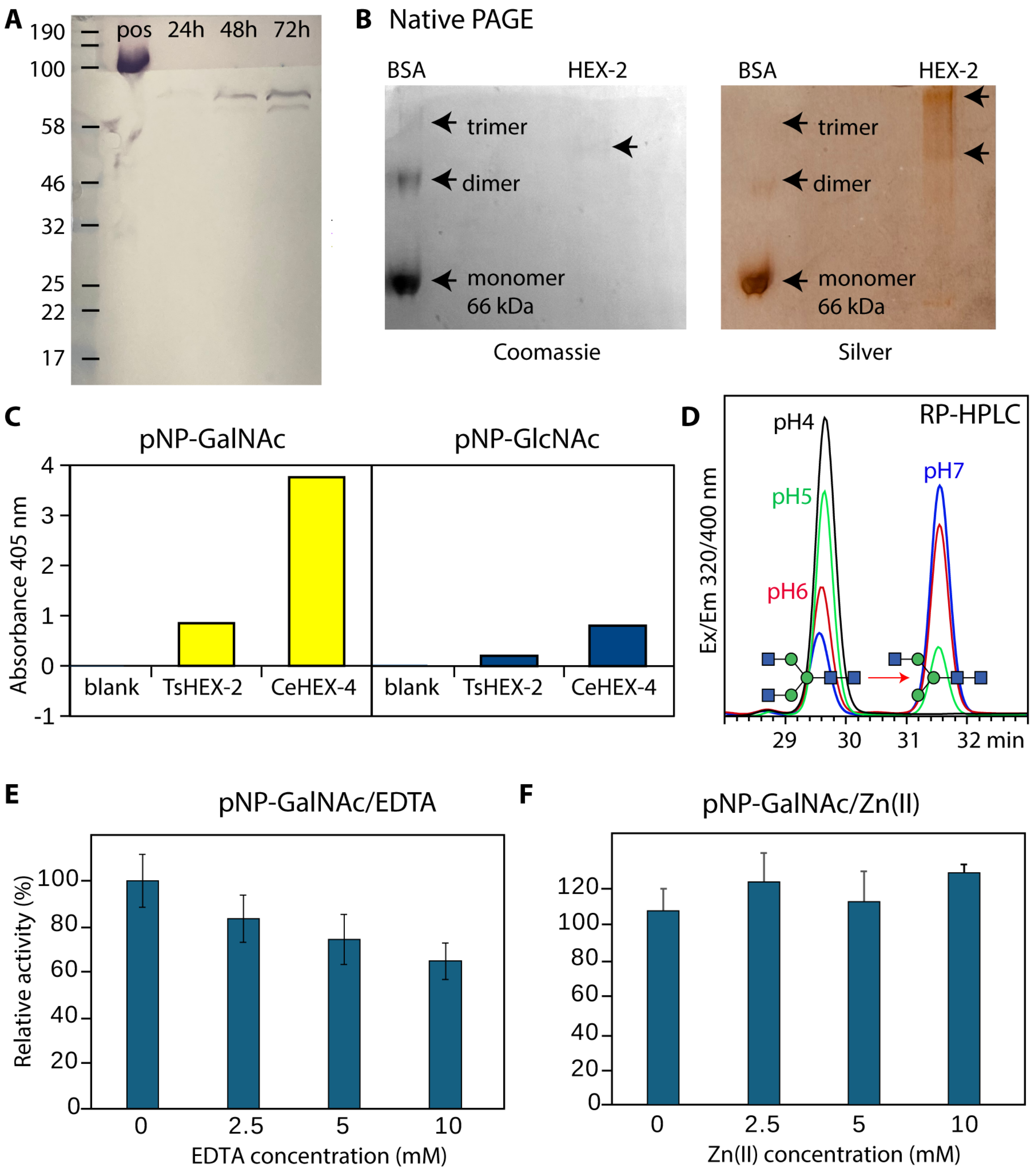

Supplementary Figure S4: (A) Expression of the recombinant secreted 'long' form of *T. suis* HEX-2 (expected M_r 63 kDa; N-terminal His/FLAG-tag) as detected by anti-FLAG Western blotting of *Pichia* culture supernatants after 24, 48 or 72 hours of induction. (B) Native gel electrophoresis of bovine serum albumin (BSA) and *T. suis* HEX-2 followed by either Coomassie or silver staining suggests that HEX-2 exists as in multimeric forms; BSA has monomeric/dimeric/trimeric molecular masses of 66, 132 and 198 kDa, whereas the dimeric form of HEX-2 would have a mass of 150 kDa as compared to the 75 kDa observed on the reducing/denaturing SDS-PAGE and Western blot. (C) Activity towards pNP-β-GalNAc and pNP-β-GlcNAc of *T. suis* HEX-2 and *C. elegans* HEX-4 expressed in *Pichia pastoris*; the turnover of the aryl GalNAc substrate is some three- or fourfold higher than that of the aryl GlcNAc in keeping with data on other subfamily 1 GH20 hexosaminidases. (D) Activity of *T. suis* HEX-2 at different pH values towards GnGn-PA; the appearance of the GnM-PA product of later RP-HPLC elution time (31.5 mins; see also ***Figure 4*** of the main text) is most pronounced at pH 6 and 7, in keeping with the optimum found with the aryl GalNAc substrate (see ***Figure 3*** of the main text). (E and F) Activity towards pNP-β-GalNAc of *T. suis* HEX-2 in the absence or presence of up to 10 mM EDTA or $ZnCl_2$, indicating minimal effect of divalent cations.

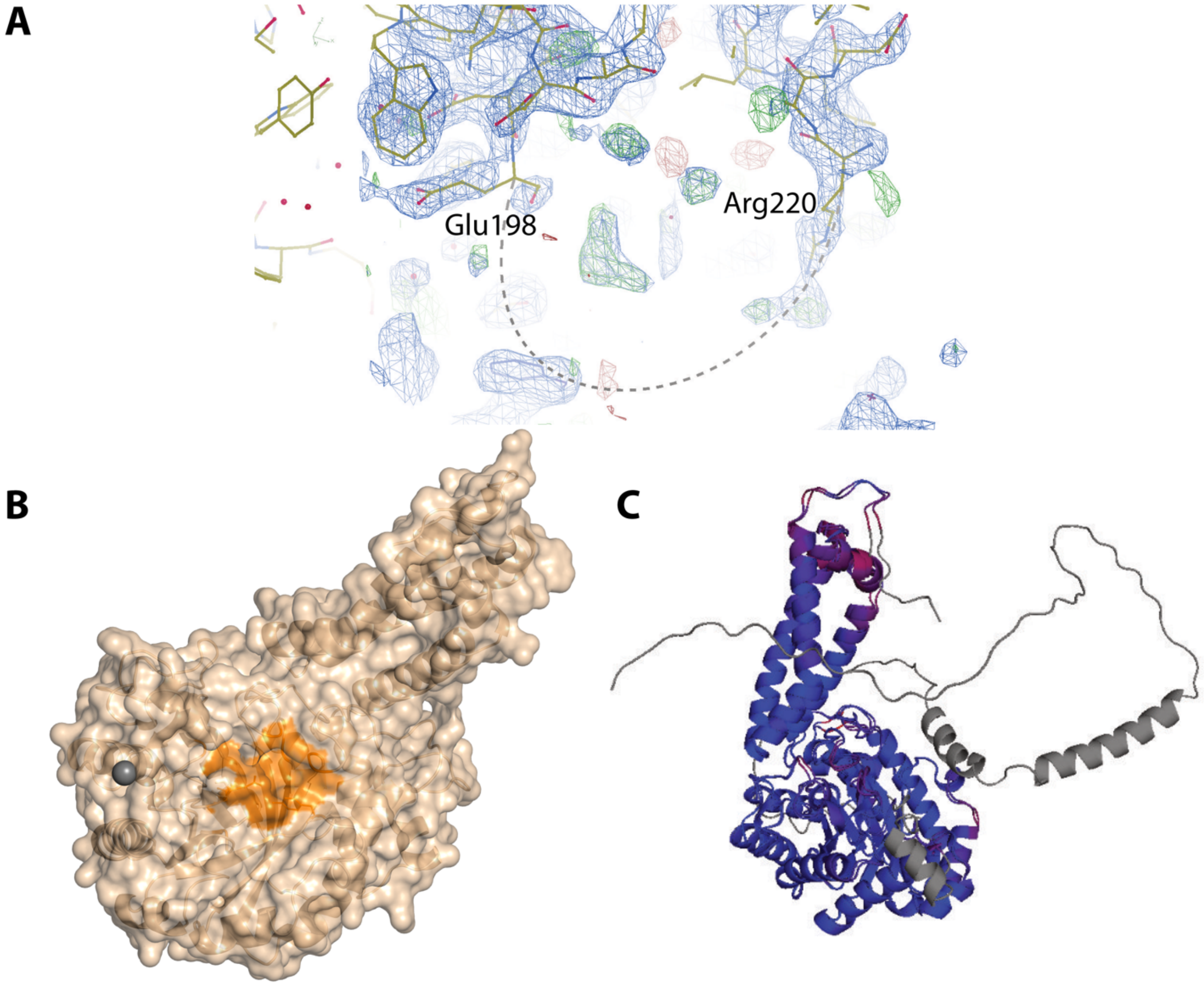

Supplementary Figure S5: (A) Electron density maps in the disordered area of the *T. suis* HEX-2 structure, visualized with Coot (version 0.9.8.3). Difference map Fo-DFc map is represented in green (positive) and red (negative) at 3 sigmas whilst 2Fo-DFc is represented in blue at 1 sigma (0.23 $eÅ^{3}$). (B) Cartoon and surface representation of crystal structure of T. suis HEX-2 at 2.55 Å. The grey ball represents the Zn^{2+} ion, the binding site pocket is colored in orange with presumed catalytic amino acids displayed in sticks. (C) Comparison of the *T. suis* HEX-2 X-ray structure and the Alpha Fold model, coloured by RMSD (blue specifying the minimum pairwise RMSD and red indicating the maximum, unaligned residues are coloured grey), visualized with pymol (free version 2.5.0, Schrodinger LLC). See also ***Figures 6 and 7*** of the main text for other visualisations.

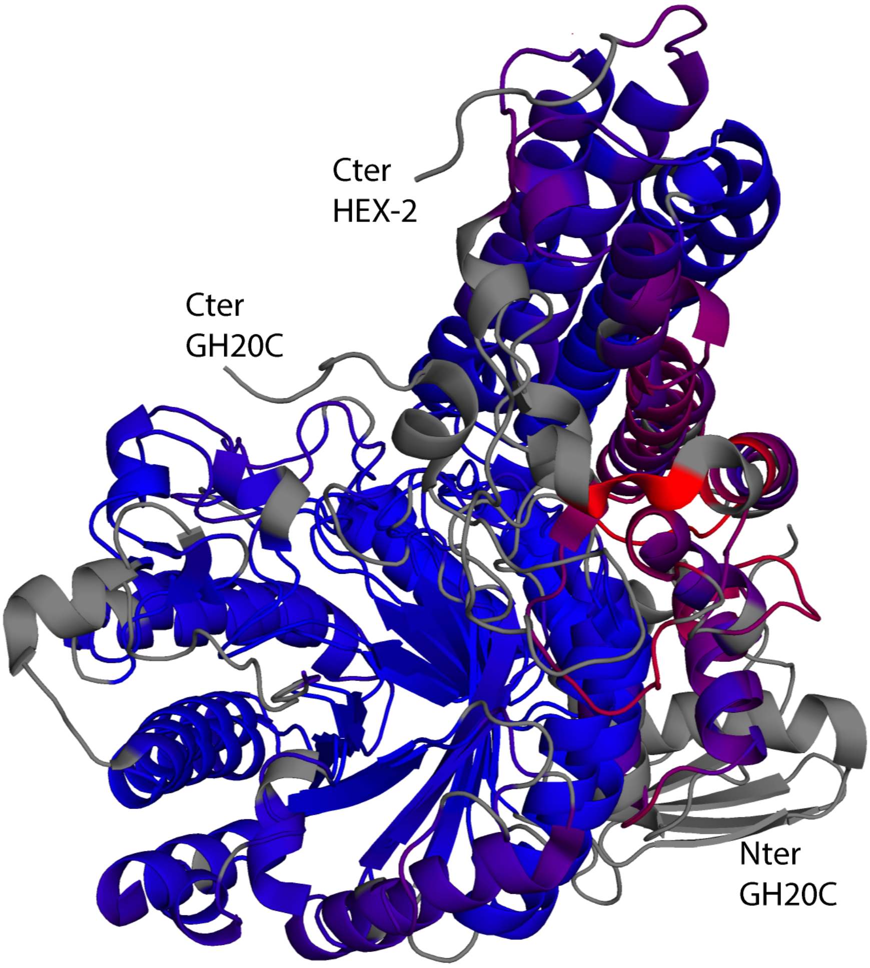

Supplementary Figure S6: Superimposition of *T. suis* HEX-2 (8QK1) and *S. pneumoniae* GH20C (5A6J) monomeric crystal structures coloured by RMSD as visualized with pymol (free version 2.5.0, Schrodinger LLC). The distances between aligned C-alpha atom pairs are stored as B-factors of these residues, which are coloured by a spectrum ranging from blue specifying the minimum pairwise RMSD and red indicating the maximum. Unaligned residues are coloured grey. The barrels containing the binding pocket superimpose well, but the N- and C-termini differ (in this view, the N-terminus of HEX-2 is hidden under the barrel). Sequence identity of HEX-2 and GH20C is 29%. See also ***Figure 6*** of the main text for other visualisations.
